# Dab2 (Disabled-2), an adaptor protein, regulates self-renewal of hair follicle stem cells

**DOI:** 10.1038/s42003-024-06047-2

**Published:** 2024-05-03

**Authors:** Sayoni Roy, Darshan Mehta, Akshay Paradkar, Gopal Chovatiya, Sanjeev K. Waghmare

**Affiliations:** 1 Stem Cell Biology Group, Waghmare Lab, Cancer Research Institute, Advanced Centre for Treatment Research and Education in Cancer (ACTREC), Tata Memorial Centre, Kharghar, Navi Mumbai, 410210 Maharashtra India; 2 Homi Bhabha National Institute, Training School Complex, Anushakti Nagar, Mumbai, 400085 India

**Keywords:** Skin stem cells, Self-renewal

## Abstract

Disabled 2 (Dab2), an adaptor protein, is up regulated in the hair follicle stem cells (HFSCs); however, its role in any tissue stem cells has not been studied. In the present study, we have reported that Dab2 conditional knockout (Dab2-cKO) mice exhibited a delay in the HF cycle due to perturbed activation of HFSCs. Further, Dab2-cKO mice showed a reduction in the number of HFSCs and reduced colony forming ability of HFSCs. Dab2-cKO mice showed extended quiescence of HFSCs concomitant with an increased expression of Nfatc1. Dab2-cKO mice showed a decreased expression of anti-aging genes such as Col17a1, decorin, Sirt2 and Sirt7. Dab2-cKO mice did not show full hair coat recovery in aged mice thereby suggesting an accelerated aging process. Overall, we unveil for the first time, the role of Dab2 that regulate activation and self-renewal of HFSCs.

## Abstract (web summary)

The adaptor protein Disabled 2 (Dab2) regulates the activation and self-renewal of hair follicle stem cells via canonical Wnt pathway. Importantly, Dab2 is also involved in the ageing process.

## Introduction

The HF represents an excellent model, to study mechanisms regulating adult stem cells quiescence and activation. HFs undergo a continuous cycle of growth, regression, and resting known as anagen, catagen, and telogen, respectively, during the mammalian hair cycle^1^. HF regeneration is driven by HFSCs located at the follicular bulge region and their progenies present in the secondary hair germ (sHG). During the telogen-to-anagen transition, quiescent HFSCs located in the hair germ respond to dermal papillae (DP) signals and gets primed, followed by activation of bulge HFSCs. Upon activation, HFSCs exit the bulge, proliferate, and differentiate to form the various lineages of the epidermal compartment. HFSCs help in the repair of the epidermis upon an injury^2–4^. In addition to maintaining the normal hair growth cycle and hair regeneration, HFSCs also regulates the ageing process in HFs. HFSC reservoir depletion, functional decline, and extended quiescence result in prominent ageing characteristics such as graying, thinning, and loss of hair. Extended telogen phases in aged HFs is a reflection of a declining capacity of HFSCs to get activated and initiate a new hair cycle^5^. HFSC activation is regulated by Wnt, BMP, Notch, and Shh pathways. Deregulation of Wnt signaling leads to perturbed HFSCs activation^6^. Loss of Sfrp1 results in increased HFSCs proliferation and accelerated HF cycle, leading to depletion of HFSCs population at PD21-PD28^7^. Dkk1 overexpression in the skin perturbs HF formation^8^. Upregulation of Wnt7b facilitates HF growth while its ablation delays HFSC activation during telogen-anagen transition^9,10^. Similarly, overexpression or transient activation of β-catenin and Lef1 in HFSCs or basal epidermis facilitates HF regeneration and de novo HF formation^11,12^. On the contrary, conditional ablation of β-catenin, or transgenic expression of a truncated form of Lef1 perturbs HF formation^13,14^. In the absence of Wnt ligands, excessive cytoplasmic β-catenin is degraded by the destruction complex comprising Axin, adenomatosis polyposis coli (APC), protein phosphatase 2A (PP2A), glycogen synthase kinase 3β (GSK3β) and casein kinase 1α (CK1α)^15,16^. The binding of Wnt ligands to a Frizzled receptor and LRP5/6 co-receptor results in the recruitment of Disheveled (Dvl) to the plasma membrane resulting in destabilization of the destruction complex, which in turn causes β-catenin stabilization and activation of the canonical Wnt signaling^17^.

Canonical Wnt signaling is modulated by various extracellular and intracellular regulators. Dab2 is a cytosolic adaptor protein which is involved in clathrin-mediated endocytosis of receptors and cargo trafficking in eukaryotes. The N-terminal PTB/PID and the C-terminal PRD domains enable Dab2 to function as an adaptor protein^18^. Dab2 interacts with various Wnt pathway components to regulate its activation. Dab2 interacts with Dvl3 via its PTB domain to inhibit Wnt-3A-mediated signaling activation^19^. Interaction of Dab2 with Axin blocks Axin-protein phosphatase 1 (PP1) association, thereby inhibiting Axin dephosphorylation and attenuating Wnt/β-catenin signaling^20^. Dab2 alters LRP6’s internalization fate by sequestering it in the clathrin-dependent endocytic route and inhibits canonical Wnt signaling^21^. Dab2 is an important regulator of embryonic development. Dab2 is essential for establishing the correct number of cardiomyocytes in the developing heart in zebrafish embryos^22^. Dab2 knockdown affects colony formation and hinders mesoderm differentiation of ESCs by disrupting ESCs cell–cell adhesion^23^. Dab2 deletion results in a reduced number of apical coated pits and vesicles and compromised function of kidney proximal tubule cells^24^. Dab2 is highly expressed in epithelial cells and maintains epithelial apical-basal polarity^25^. Though Dab2 is widely expressed in a number of adult tissues such as the heart, kidney, ovary, liver, mammary glands, intestine, and uterus^26^, its role in adult tissue maintenance remains obscure.

In this study, we report that K14Cre-specific Dab2 deletion results in HFSCs pool reduction, extended quiescence, decreased activation potential, and compromised self-renewal capacity of HFSCs. Dab2 loss shows downregulation of ageing-related genes and compromised hair regeneration capacity, thereby resulting in accelerated ageing. Overexpression of Dvl2 in Dab2-cKO keratinocytes results in partial restoration of β-catenin activity and colony-forming capacity of keratinocytes. Thus, in this study, we have shown a context-dependent role of Dab2 in HFSCs in which Dab2 primarily stabilizes Dvl2 and promotes canonical Wnt signaling activation which in turn initiates HF cycle entry and progression.

## Results

### Dab2 is dynamically expressed during the HF cycle

**Fig. 1:**
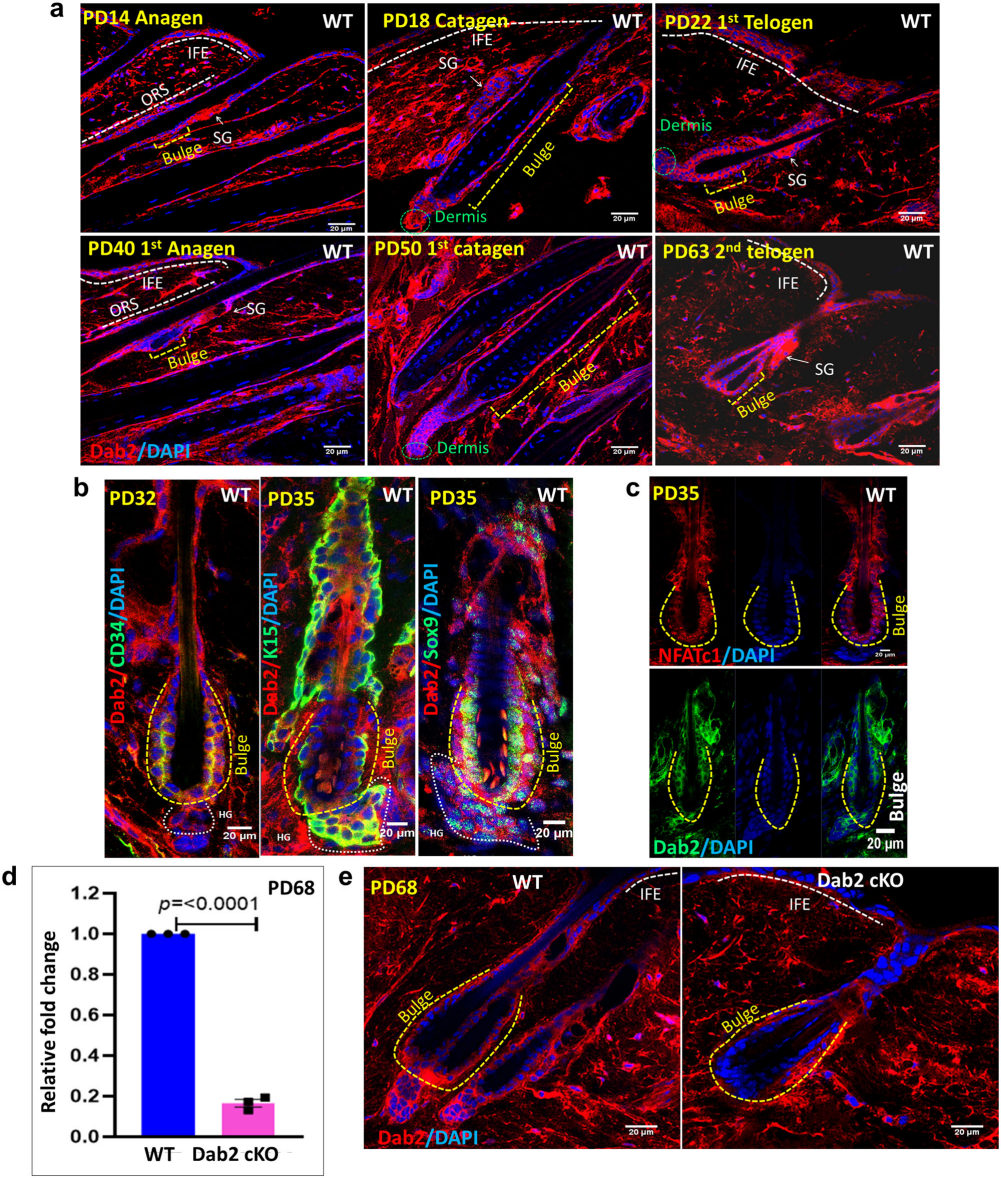
Expression of Dab2 during various stages of HF morphogenesis and first adult HF cycle. **a** Dab2 expression in WT during hair morphogenesis at PDs 14, 18, 22, and first adult hair cycle at PDs 40, PD50, and PD63, *n* = 3 independent biological replicates. **b** Co-staining of Dab2 with CD34, K15, and Sox9 at indicated days, *n* = 3 independent biological replicates. **c** Co-staining of Dab2 with NFATc1 at PD35, *n* = 3 independent biological replicates. **d** qPCR analysis of Dab2 expression in WT and Dab2-cKO in epidermis, *n* = 3 independent biological replicates for each genotype, mean ± SEM, *p < 0.05, **p < 0.01, ***p < 0.001, ****p < 0.0001 obtained by students *t*-test. **e** IFA of Dab2 in WT and Dab2-cKO cryopreserved dorsal skin sections, *n* = 3 independent biological replicates for each genotype.

To understand the Dab2 expression pattern during HF cycling, we performed an immunofluorescence assay (IFA) of Dab2 at various postnatal days (PDs). During morphogenesis, at PD7 (mid anagen) and PD14 (late anagen), Dab2 expression was observed in the entire HF including the infundibulum, isthmus, suprabulbar region, and bulb region (Supplementary Fig. 1a and Fig. 1a). At PD18 (catagen), Dab2 was observed in the retracting epithelial strand and the DP. At PD22, Dab2 was observed in the junctional zone, bulge region, HG, and DP region. As anagen progressed during the first adult hair cycle, at PD40 (anagen IV), Dab2 was again observed in the entire HF, including the infundibulum, isthmus, suprabulbar region, and bulb region. The pattern of Dab2 expression during the first catagen (PD45) and second telogen (PD55) was similar to that observed during the catagen and telogen phases of HF morphogenesis. Moreover, Dab2 expression was observed in the interfollicular epidermis (IFE) region, sebaceous gland (SG) and DP at all stages of the hair cycle (Fig. 1a). Co-staining of Dab2 with markers such as CD34, K15, Sox9, and NFATc1 further confirmed the fact that Dab2 is expressed both in the bulge and HG region of the HF (Fig. 1b, c). It was noted that Dab2 expression increased in the lower bulge and HG region, marked by K15, at telogen-anagen transition (PD35), indicating that Dab2 might play a significant role in the HF cycle initiation. Dab2^fl/fl^ was crossed with K14CreER^+/−^ to generate K14-specific inducible Dab2 conditional knockout mice (Dab2-cKO) by tamoxifen injection from PD22–26 (Supplementary Fig. 1b). We used the ROSA-YFP reporter mice to standardize the tamoxifen dosage for activation of Cre in K14+ cells. The YFP expression in IFE and HF regions suggested that the injected dose of tamoxifen was sufficient to induce Cre activity (Supplementary Fig. 1c). qPCR analysis revealed ~80% reduction in Dab2 mRNA expression in Dab2-cKO mice. IFA staining of Dab2 also confirmed the Dab2 deletion in the HF (Fig. 1d, e).

### Dab2 controls hair cycle entry

**Fig. 2:**
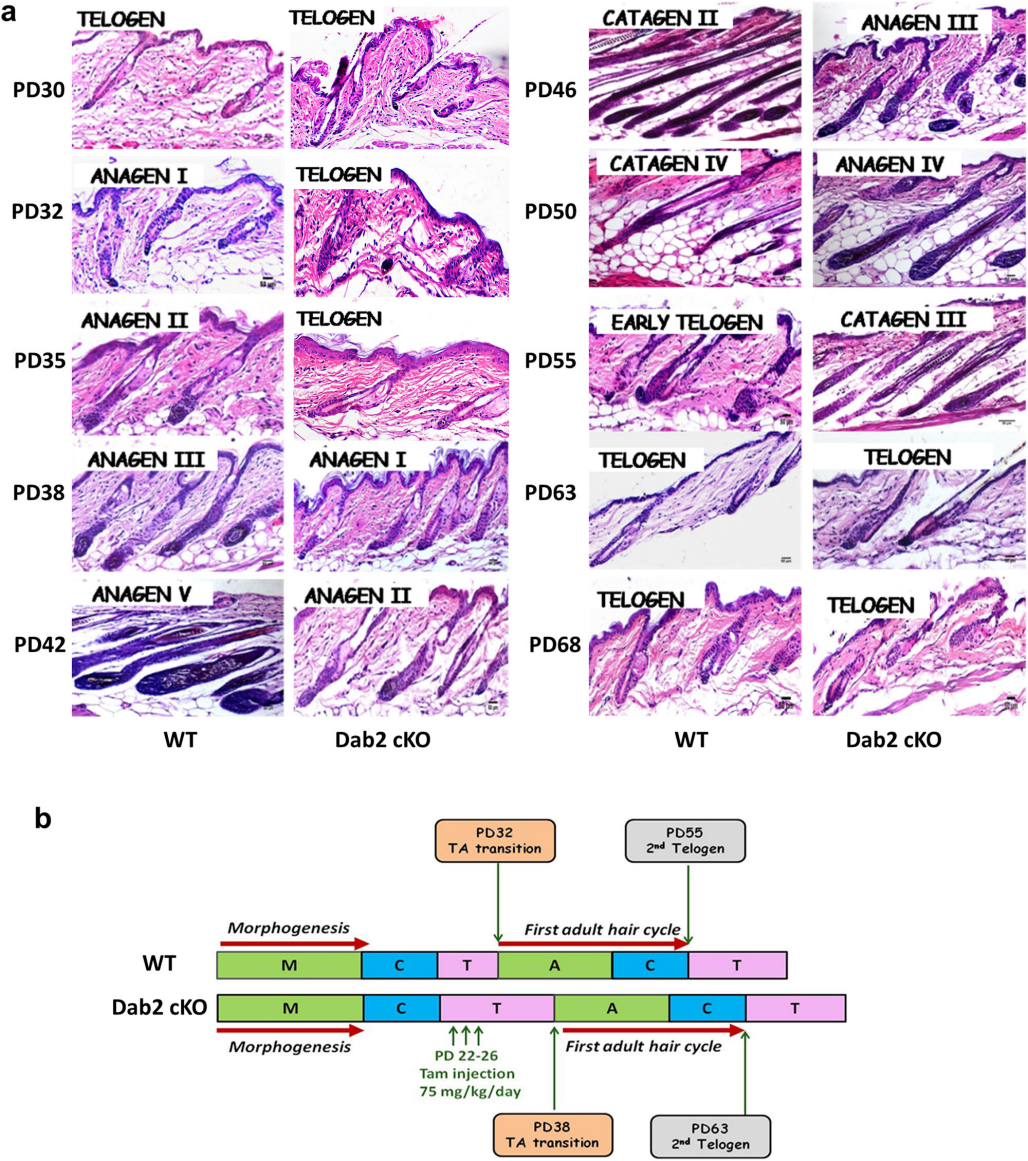
Histological analysis of first adult HF cycle progression. **a** H&E staining of WT and Dab2-cKO formalin-fixed dorsal skin sections at various PDs during the first adult hair cycle (PDs—30, 32, 35, 38, 42, 46, 50, 55, 63, 68), *n* = 3 independent biological replicates for each PD in each genotype. **b** Schematic diagram showing HF cycling pattern in WT and Dab2-cKO as observed by H&E staining mentioned above.

To examine if Dab2 deletion in skin epithelium affects the adult hair cycle, we compared the hair cycle stages between age-matched females of WT and Dab2-cKO. H&E staining revealed that both WT and Dab2-cKO were present at the telogen phase at PD30. However, as the cycle progressed, a delay in the transition from the first telogen to the subsequent anagen was observed in Dab2-cKO (Fig. 2a, b). In WT, the telogen-to-anagen transition began at PD32, followed by progression to anagen II at PD35 and anagen III at PD38. On the contrary, Dab2-cKO HFs remained in the telogen phase at PD32 and PD35. The telogen-anagen transition began only at PD38 in Dab2-cKO. At PD50, while the WT reached the catagen IV phase, the Dab2-cKO reached the anagen IV phase. Subsequently, the WT reached the telogen phase at PD55 while the Dab2-cKO reached telogen at PD63. At PD68, both WT and Dab2-cKO were observed to be at the second telogen phase (Fig. 2a). Thus, Dab2 loss leads to a delay in first adult HF cycle entry and HF cycle progression.

### Dab2 regulates the activation and proliferation dynamics of HFSCs

**Fig. 3:**
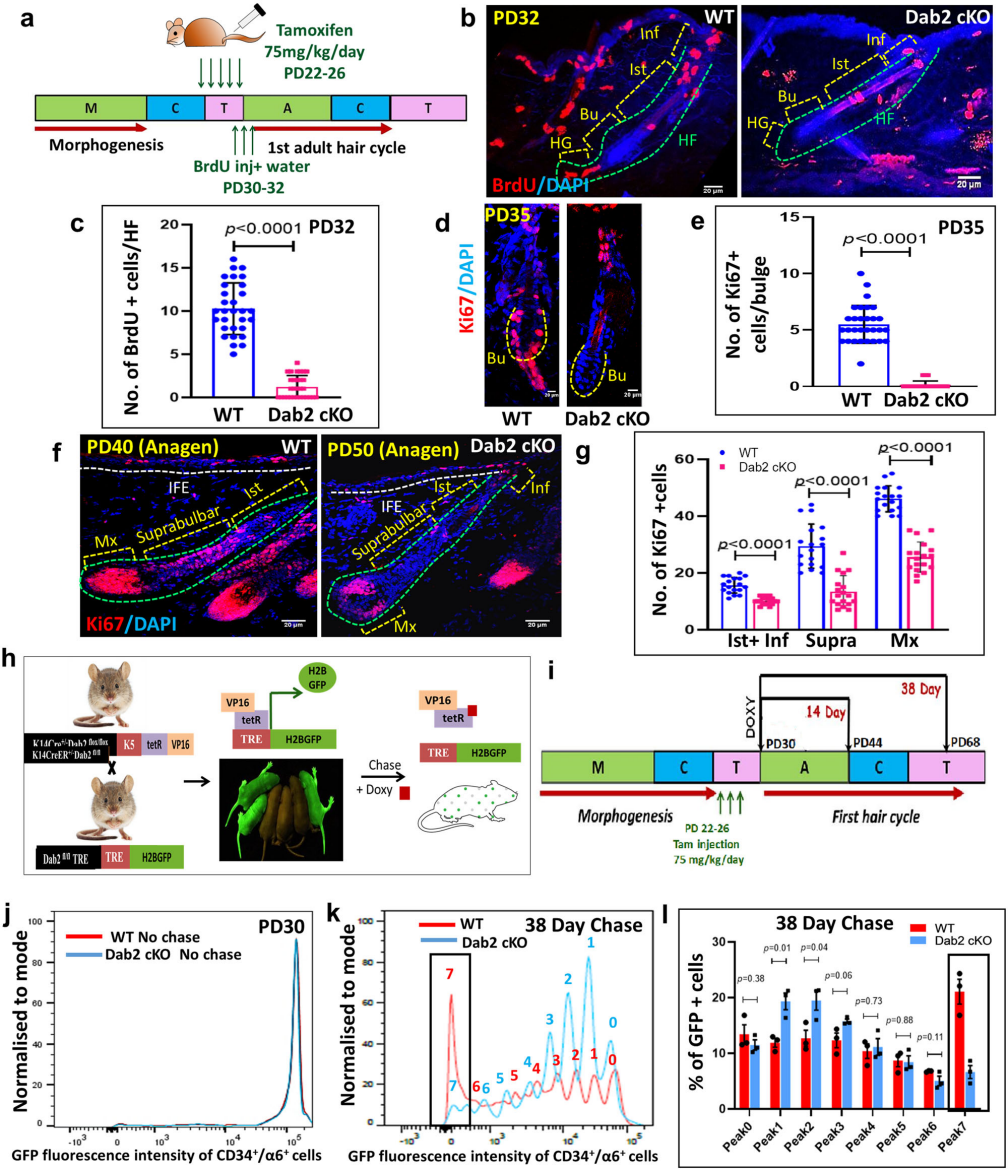
Effect of Dab2 deletion on activation and proliferation dynamics of HFSCs. **a** Strategy to perform the BrdU proliferation assay at PD32. **b** IFA of BrdU expression in WT and Dab2-cKO HFs at PD32, *n* = 3 independent biological replicates for each genotype. **c** Graph representing BrdU+ cells in HFs at PD32 in WT and Dab2-cKO mice, *n* = 3 independent biological replicates for each genotype, mean ± SD, *p < 0.05, **p < 0.01, ***p < 0.001, ****p < 0.0001 obtained by students *t*-test. **d** IFA showing Ki67 expression in WT and Dab2-cKO bulge at PD35, *n* = 3 independent biological replicates for each genotype. **e** Graph representing Ki67+ cells in WT and Dab2-cKO bulge at PD35, *n* = 3 independent biological replicates for each genotype, mean ± SD, *p < 0.05, **p < 0.01, ***p < 0.001, ****p < 0.0001 obtained by students *t*-test. **f** IFA showing Ki67 expression in WT anagen (PD40) and Dab2-cKO anagen (PD50) follicles, *n* = 3 independent biological replicates for each genotype. **g** Graph representing Ki67+ cells in various regions of HF in WT anagen and Dab2-cKO anagen follicles at indicated days, *n* = 3 independent biological replicates for each genotype, mean ± SD, *p < 0.05, **p < 0.01, ***p < 0.001, ****p < 0.0001 obtained by students *t*-test. **h** Schematic diagram showing crosses done to obtain K14Cre^+/−^:H2BGFP:K5Tta:Dab2^fl/fl^ background. **i** Schematic diagram showing strategy adopted to perform doxy chases for indicated days. **j** FACS histogram showing GFP intensity peaks of unchased WT and Dab2-cKO mice at PD30. **k** GFP intensity peaks showing H2BGFP dilution in FACS sorted HFSCs after doxy chase from PD30 to PD68, *n* = 3 independent biological replicates for each genotype. **l** Graph showing the percentage of GFP+ cells in each GFP intensity peak. Peak 0 represents cell fractions with the highest GFP intensities, Peak 7 represents cell fractions with lowest GFP intensities, *n* = 3 independent biological replicates for each genotype, mean ± SEM, *p < 0.05, **p < 0.01, ***p < 0.001, ****p < 0.0001 obtained by students *t*-test. Inf Infundibulum, Ist Isthmus, Bu Bulge, Mx Matrix, Supra Suprabulbar region.

To investigate whether Dab2 deletion affects the proliferative potential of HFSCs, we performed the BrdU proliferation assay at PD32 which revealed that BrdU uptake was significantly reduced in Dab2-cKO HFs. Dab2-cKO exhibited ~1 BrdU+ cells/HF, while WT exhibited ~10 BrdU+ cells/HF (Fig. 3a–c). Similarly, there was ~85% reduction in the number of Ki67+ cells/HF in Dab2-cKO (Supplementary Fig. 2a, b). The HF regions, such as the infundibulum region, isthmus region, bulge region, and hair germ, were considered for analyzing BrdU+ and Ki67+ cells. As the HF cycle progressed, at PD35, the WT mice exhibited ~6 Ki67+ cells/bulge as compared to Dab2-cKO, which exhibited ~0.1 Ki67+ cells/bulge (Fig. 3d, e). Furthermore, Ki67 staining analysis at anagen phases of WT (PD40) and Dab2-cKO (PD50) revealed significantly reduced proliferation in the Dab2-cKO HFs (Fig. 3f, g). Co-staining of Ki67 and K14 also revealed that WT exhibited ~12 Ki67+ cells/~150 µm region of IFE, while Dab cKO mice exhibited ~0.1 Ki67 cells/~150 μm region of IFE (Supplementary Fig. 2c, d). These results clearly indicate that loss of Dab2 leads to reduced proliferative capacity in the entire HF and the IFE. Moreover, loss of Dab2 not only affects the initial activation potential of HFSCs during the telogen-to-anagen transition stage but also affects the proliferative capacity of keratinocytes in the growth (anagen) phase. To further confirm the effect of Dab2 on the proliferation dynamics of HFSCs, we used the Tet-off H2BGFP/K5tTA system where the expression of GFP can be shut down by feeding the mice with doxycycline (doxy) feed for a specific time period known as the chase period (Fig. 3h, i). In the unchased mice, we observed equal fluorescence intensities of H2BGFP in both the WT and Dab2-cKO mice at PD30, suggesting an equal efficiency of the initial labeling (Fig. 3j). To assess the initial rate of HFSCs proliferation, doxy chase was done for 2 weeks from PD30 till PD44 (14 days chase). We observed a twofold dilution of GFP intensities with each division in both WT and Dab2-cKO (Supplementary Fig. 3a). An increased cell fraction (~50%) with lower H2BGFP fluorescence (peaks 4–7) was observed in WT as compared to Dab2-cKO which exhibited ~33% GFP+ cells in peaks 4–7 thereby indicating that WT HFSCs divides more than Dab2-cKO during 14-day chase (Supplementary Fig. 3b, c).

To assess the rate of proliferation throughout the entire hair cycle, we performed a doxy chase from PD30-PD68 (38-day chase). We observed a twofold dilution of GFP intensities with each division in both WT and Dab2-cKO (Supplementary Fig. 3d). In WT, ~21% of GFP+ cells were present in peak 7 (Lowest GFP intensity) as compared to Dab2-cKO, which exhibited only ~7% GFP+ cells in peak 7 (Fig. 3k, l) indicating that WT HFSCs undergoes multiple rounds of divisions resulting in the sequential serial dilution of H2BGFP. On the contrary, Dab2-cKO HFSCs are relatively slow cycling throughout the first adult hair cycle.

### Dab2 is essential for maintaining HFSCs pool at first telogen

**Fig. 4:**
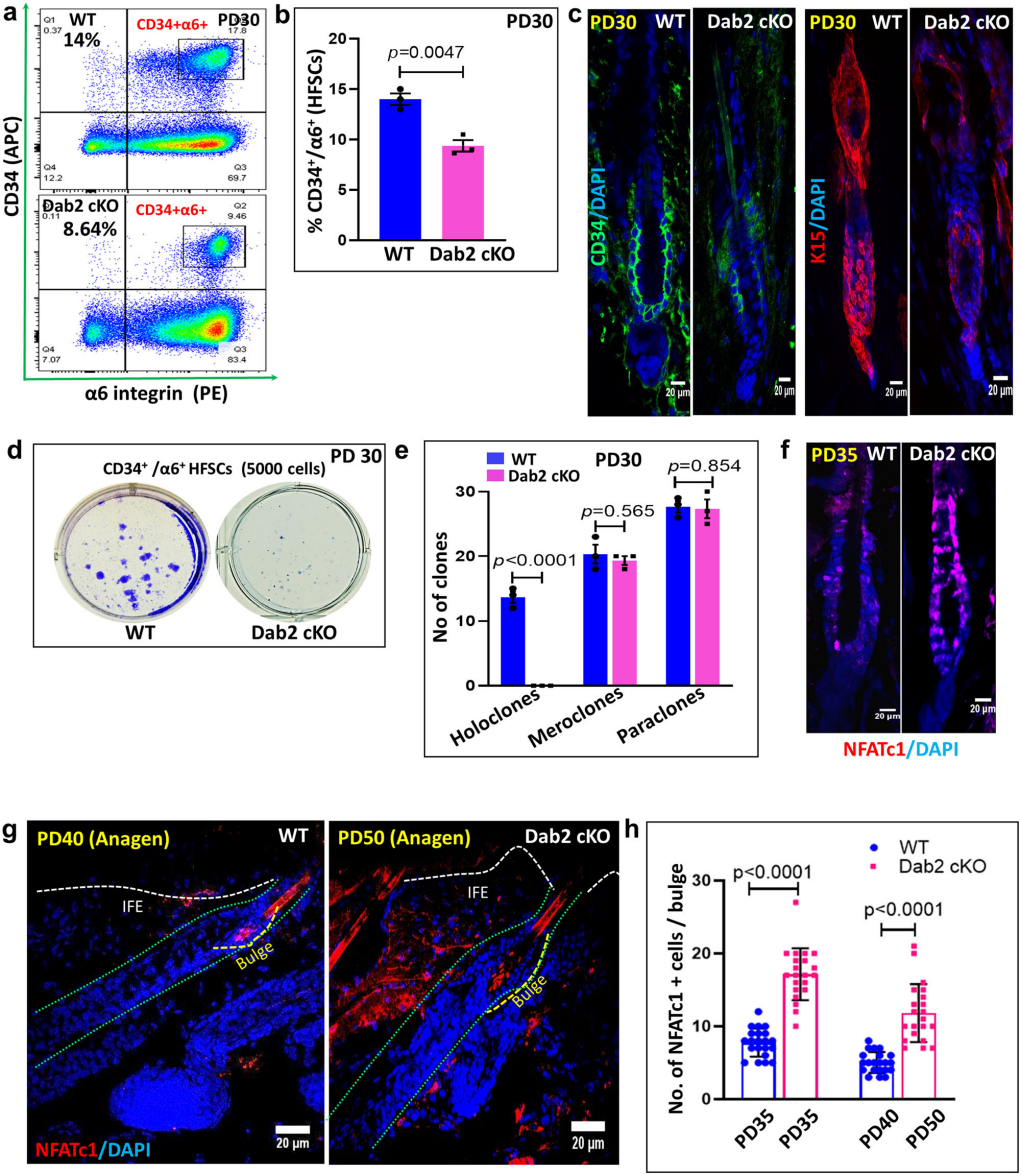
Effect of Dab2 deletion on self-renewal capacity and quiescence. **a** FACS analysis showing CD34^+^/α6-integrin^+^ HFSCs population at PD30 in WT and Dab2-cKO mice, *n* = 3 independent biological replicates for each genotype. **b** Graph showing percentage of CD34^+^/α6-integrin^+^ HFSCs population at PD30 in WT and Dab2-cKO mice, *n* = 3 independent biological replicates for each genotype, mean ± SEM, *p < 0.05, **p < 0.01, ***p < 0.001, ****p < 0.0001 obtained by students *t*-test. **c** IFA of CD34 and K15 expression in WT and Dab2-cKO mice at PD30, *n* = 3 independent biological replicates for each genotype. **d** Colonies formed by FACS sorted CD34^+^/α6-integrin^+^ HFSCs from PD30 WT and Dab2-cKO mice when cultured for 3 weeks, *n* = 3 independent replicates for each genotype, mean ± SEM, *p < 0.05, **p < 0.01, ***p < 0.001, ****p < 0.0001 obtained by students *t*-test. **e** Graph showing no. of holoclones, meroclones and paraclones formed by FACS sorted WT and Dab2-cKO HFSCs at PD30, *n* = 3 independent biological replicates for each genotype, mean ± SEM, *p < 0.05, **p < 0.01, ***p < 0.001, ****p < 0.0001 obtained by students *t*-test. **f** IFA of NFATc1 expression in WT and Dab2-cKO mice at PD35, *n* = 3 independent biological replicates for each genotype. **g** IFA of NFATc1 in WT anagen (PD40) and Dab2-cKO anagen (PD50), *n* = 3 independent biological replicates for each genotype. **h** Graph showing NFATc1+ cells in WT and Dab2-cKO bulge at indicated days, *n* = 3 independent replicates for each genotype, mean ± SD, *p < 0.05, **p < 0.01, ***p < 0.001, ****p < 0.0001 obtained by students *t*-test.

We next examined the role of Dab2 in the maintenance of the HFSC pool at PD30, when both WT and Dab2-cKO mice were at the first telogen phase. FACS analysis revealed a ~35% reduction in the CD34^+^/α6-integrin^+^ HFSCs population in Dab2-cKO (Fig. 4a, b). IFA revealed reduced expression of CD34 and K15 in the Dab2-cKO bulge (Fig. 4c). The colony-forming assay (CFA) revealed that Dab2-cKO HFSCs did not form holoclones. However, approximately an equal number of meroclones and paraclones were formed by both WT and Dab2-cKO HFSCs (Fig. 4d, e). Subsequently at PD35, WT had entered anagen II, but Dab2-cKO mice were still at the telogen phase. Therefore, at PD35, FACS analysis revealed ~ 45% reduction in the CD34^+^/α6-integrin^+^ HFSCs population in Dab2-cKO (Supplementary Fig. 4a, b). Dab2-cKO HFSCs formed relatively a fewer number of holoclones and meroclones (Supplementary Fig. 4c, d). Reduced expression of CD34 and K15 was observed in the Dab2-cKO bulge (Supplementary Fig. 4e). However, Sox9+ cells were significantly increased in the Dab2-cKO bulge (Supplementary Fig. 4f, g). An elevated expression of niche factors such as FGF18, Col17a1, and Decorin and reduced expression of Sirt2, Sirt7, and S100a4 was observed in Dab2-cKO HFSCs (Supplementary Fig. 5a). We also observed an increased number of NFATc1+ cells in Dab2-cKO bulge at PD35 (Fig. 4f, h). Moreover, even when Dab2-cKO mice progressed to the anagen phase at PD50, a higher number of NFATc1 cells were observed in the bulge region as compared to the WT anagen bulge (PD40) (Fig. 4g, h). Together, these data indicate that Dab2 deletion results in reduced HFSCs pool and reduced self-renewal capacity. Dab2 loss also affects the expression of NFATc1, leading to extended quiescence of HFSCs and delayed HF cycle initiation at PD35.

### Dab2 regulates the label retention and self-renewal capacity of HFSCs at 2^nd^ telogen

**Fig. 5:**
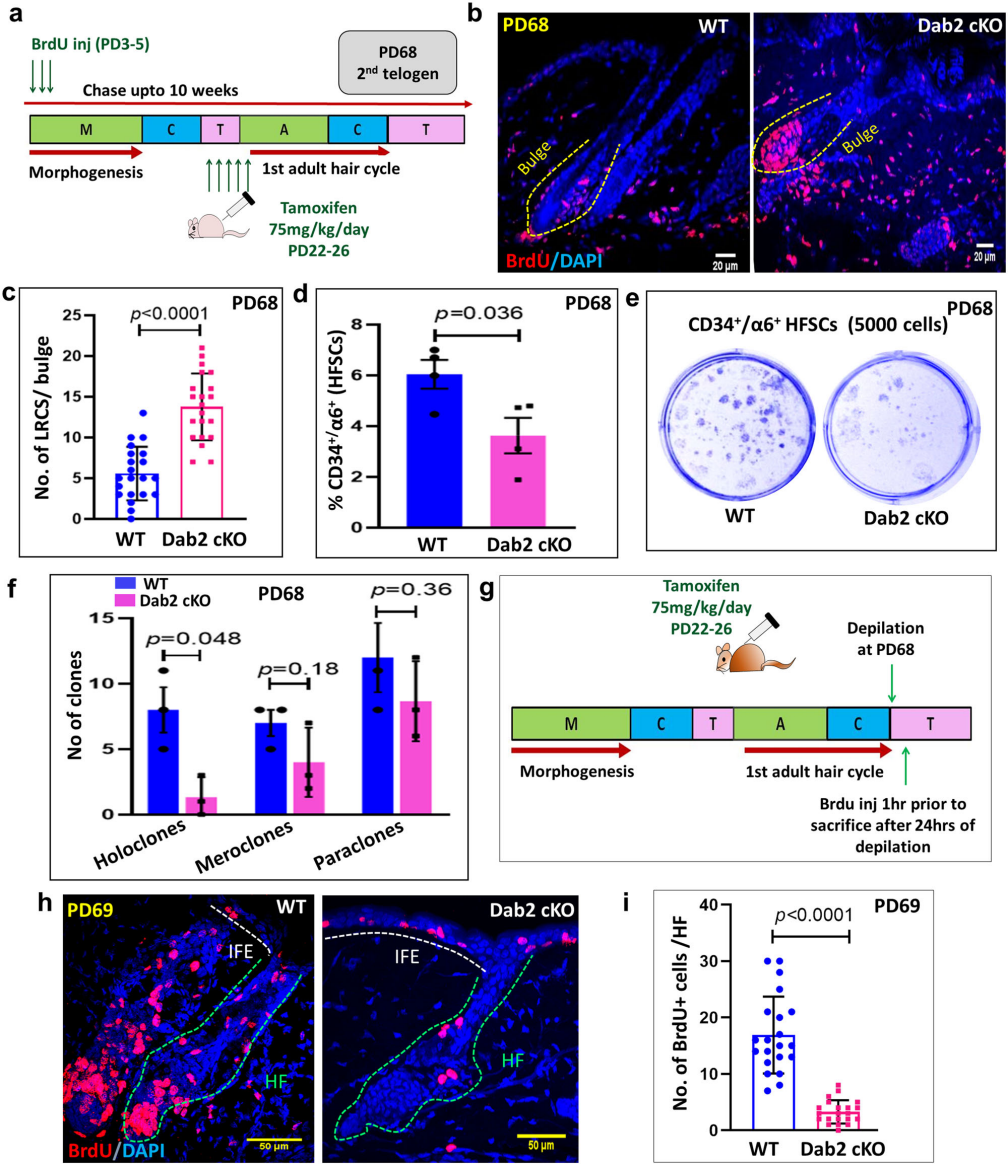
Effect of Dab2 loss on label retention capacity and activation potential of HFSCs at 2^nd^ telogen (PD68). **a** Schematic diagram showing the strategy adopted to perform BrdU label-retention assay at indicated days. **b** IFA showing BrdU expression in WT and Dab2-cKO mice after 10 weeks of BrdU chase at PD68, *n* = 3 independent biological replicates for each genotype. **c** Graph showing LRCs in WT and Dab2-cKO bulge after 10 weeks of BrdU chase at PD68, *n* = 30 follicles from three independent replicates for each genotype, mean ± SD, *p < 0.05, **p < 0.01, ***p < 0.001, ****p < 0.0001 obtained by students *t*-test. **d** Graph showing percentage of CD34^+^/α6-integrin^+^ HFSCs population at PD68 in WT and Dab2-cKO mice, *n* = 3 independent biological replicates for each genotype, mean ± SEM, *p < 0.05, **p < 0.01, ***p < 0.001, ****p < 0.0001 obtained by students *t*-test. **e** Colonies formed by FACS sorted CD34^+^/α6-integrin+ HFSCs from PD68 WT and Dab2-cKO mice when cultured for 3 weeks, *n* = 3 independent replicates for each genotype, mean ± SEM, *p < 0.05, **p < 0.01, ***p < 0.001, ****p < 0.0001 obtained by students t-test. f Graph showing no. of holoclones, meroclones and paraclones formed by FACS sorted WT and Dab2-cKO HFSCs at PD68, *n* = 3 independent biological replicates for each genotype, mean ± SEM, *p < 0.05, **p < 0.01, ***p < 0.001, ****p < 0.0001 obtained by students *t*-test. **g** Schematic diagram showing timeline and strategy adopted to perform BrdU uptake assay after depilation in WT and Dab2-cKO mice. **h** IFA showing BrdU expression in WT and Dab2-cKO mice at Day1 post-depilation (PD69). **i** Graph showing BrdU+ cells/HF in WT and Dab2-cKO mice at Day1 post-depilation (PD69), *n* = 3 independent biological replicates for each genotype, mean ± SD, *p < 0.05, **p < 0.01, ***p < 0.001, ****p < 0.0001 obtained by students *t*-test.

In order to determine whether Dab2 deletion affected the label retention capacity of HFSCs, we performed the BrdU label-retention assay (Fig. 5a). IFA of BrdU at PD6 revealed 100% BrdU labeling efficiency in HFs (Supplementary Fig. 5b). Post 10 weeks chase at PD68 (second telogen), Dab2-cKO mice exhibited ~14 BrdU+ LRCs, while the WT exhibited ~6 BrdU+ LRCs (Fig. 5b, c). At PD68, Dab2 deletion resulted in ~50% reduction in the CD34^+^/α6-integrin^+^ HFSCs population (Supplementary Fig. 5c and Fig. 5d). Dab2-cKO HFSCs formed a significantly reduced number of holoclones (Fig. 5e, f). IFA revealed reduced expression of CD34 and K15 (Supplementary Fig. 5d, e) and an increased number of NFATc+ and Sox9+ cells in the Dab2-cKO bulge (Supplementary Fig. 5f, g). To analyze the effect of Dab2 loss on the depilation-induced activation of HFSCs, we performed depilation at PD68 followed by BrdU pulse-chase analysis (Fig. 5g). On post-depilation Day1 (PD69), we observed that Dab2-cKO exhibited ~3 BrdU+ cells/HF as compared to ~16 BrdU+ cells/ HF in WT (Fig. 5h, i), indicating that loss of Dab2 resulted in perturbed activation of HFSCs post-depilation. Together, these results indicate that loss of Dab2 leads to a reduced HFSCs pool, reduced self-renewal capacity, and perturbed activation of HFSCs at the second telogen.

### Dab2 regulates ageing in murine skin

**Fig. 6:**
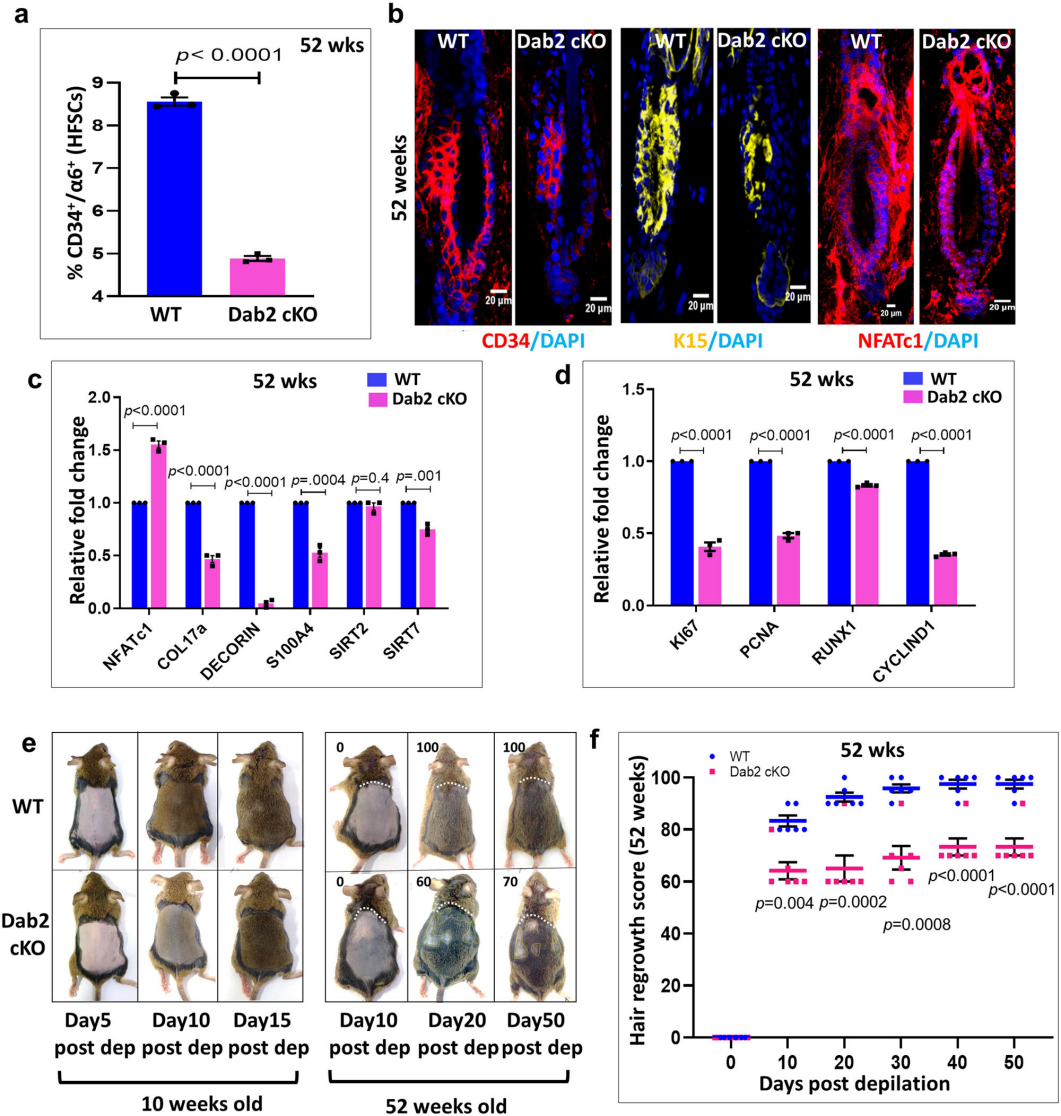
Effect of Dab2 loss on hair regrowth and ageing in murine skin. **a** Graph showing percentage of CD34+/α6-integrin+ HFSCs population in 52 weeks old WT and Dab2-cKO mice, *n* = 3 independent biological replicates for each genotype, mean ± SEM, *p < 0.05, **p < 0.01, ***p < 0.001, ****p < 0.0001 obtained by students *t*-test. **b** IFA showing CD34, K15, NFATc1 expression in 52 weeks old WT and Dab2-cKO mice, *n* = 3 independent biological replicates for each genotype. **c** qPCR analysis of niche related genes expression in 52 weeks old WT and Dab2-cKO skin, *n* = 3 independent biological replicates for each genotype, mean ± SEM, *p < 0.05, **p < 0.01, ***p < 0.001, ****p < 0.0001 obtained by students *t*-test. **d** qPCR analysis of cell-cycle related genes expression in 52 weeks old WT and Dab2-cKO skin, *n* = 3 independent biological replicates for each genotype, mean ± SEM, *p < 0.05, **p < 0.01, ***p < 0.001, ****p < 0.0001 obtained by students *t*-test. **e** The hair coats of 10 weeks old (young) and 52 weeks old (aged) WT and Dab2-cKO mice were depilated and hair regrowth was monitored till indicated days. For the aged mice group, the representative photographs of hair regrowth of WT and Dab2-cKO mice with the respective arbitrary hair regrowth scores are shown. The white line demarcates the depilated area from the rest of the area. The yellow line marks the patches where there is no hair growth. Arbitrary values from 0 to 100 based on hair shaft density was assigned, with 0 indicating no hair growth only in the depilated area and higher numbers corresponding to areas of hair regrowth in a depilated area based on photographs analyzed on the image editing tool. **f** Graph showing hair regrowth in aged WT and Dab2-cKO mice at indicated days. Arbitrary values from 0 to 100 based on hair shaft density was assigned, with 0 indicating no hair growth only in the depilated area and higher numbers corresponding to areas of hair regrowth in depilated area based on photographs analyzed on the image editing tool, *n* = 7 independent biological replicates for each genotype, mean ± SEM, *p < 0.05, **p < 0.01, ***p < 0.001, ****p < 0.0001 obtained by students *t*-test).

Next, we examined the effect of Dab2 deletion on the aged (52 weeks old) mice. The expression of Dab2 in the bulge region of aged mice was comparable to that of young (10 weeks old) mice (Supplementary Fig. 6a). Dab2 deletion resulted in ~45% decrease in the HFSCs pool in aged Dab2-cKO (Supplementary Fig. 6b and Fig. 6a). Moreover, reduced expression of CD34, K15 and increased number of NFATc1+ cells were observed in aged Dab2-cKO bulge (Fig. 6b). These results indicate that loss of Dab2 results in reduced number of HFSCs and extended quiescence in aged mice similar to that observed in young mice. Furthermore, a significant reduction of anti-ageing niche factors such as Col17a, Decorin, S100a4, Sirt2, and Sirt7 and proliferation markers such as Ki67, PCNA, and cyclin D1 was observed in aged Dab2-cKO mice (Fig. 6c, d). Dab2 deletion also resulted in a significant reduction in the expression of Wnt ligands such as Wnt1, Wnt3a, Wnt4 (Supplementary Fig. 6c) and a significant increase in the expression of Wnt inhibitors such as Sfrp4, Dkk1, Dkk2, Dkk3 in aged Dab2-cKO mice (Supplementary Fig. 6d). Ageing causes HFSCs to become more quiescent and relatively difficult to activate as compared to young mice. To investigate whether loss of Dab2 led to a more prolonged delay in HFSCs activation and hair regrowth in aged mice as compared to young mice, we performed depilation studies in both young and aged WT and Dab2-cKO mice at the same time. Hair coats on the dorsal skin of young mice were depilated using wax and hair regrowth was monitored. The results indicated that there was a delay in hair coat recovery in young Dab2-cKO mice. At post-depilation day 5, the WT mice showed pigmented skin, while the Dab2-cKO mice skin was still pink indicative of the telogen phase. At post-depilation day 10, while there was a full hair coat recovery in WT, the Dab2-cKO mice showed signs of pigmentation and hair growth initiation. Although full hair coat recovery was observed at post-depilation day 15 in Dab2-cKO mice, hair regrowth was slower and less robust as compared to WT (Fig. 6e). In aged WT mice, while 100% hair coat recovery was observed post-depilation day 20 in WT mice, only 60% hair coat recovery was observed in Dab2-cKO mice. Moreover, only 70% of hair coat was recovered in Dab2-cKO mice even after post-depilation day 50 (Fig. 6e, f). Hair regrowth was considerably slower in both aged WT and aged Dab2-cKO mice as compared to their young counterparts. However, the effect of Dab2 loss on hair coat recovery was more prominent in aged mice as compared to their young counterparts. Together these data indicate that loss of Dab2 accelerates the ageing process in murine hair follicles.

### Dab2 ablation alters canonical Wnt signaling in HFSCs

**Fig. 7:**
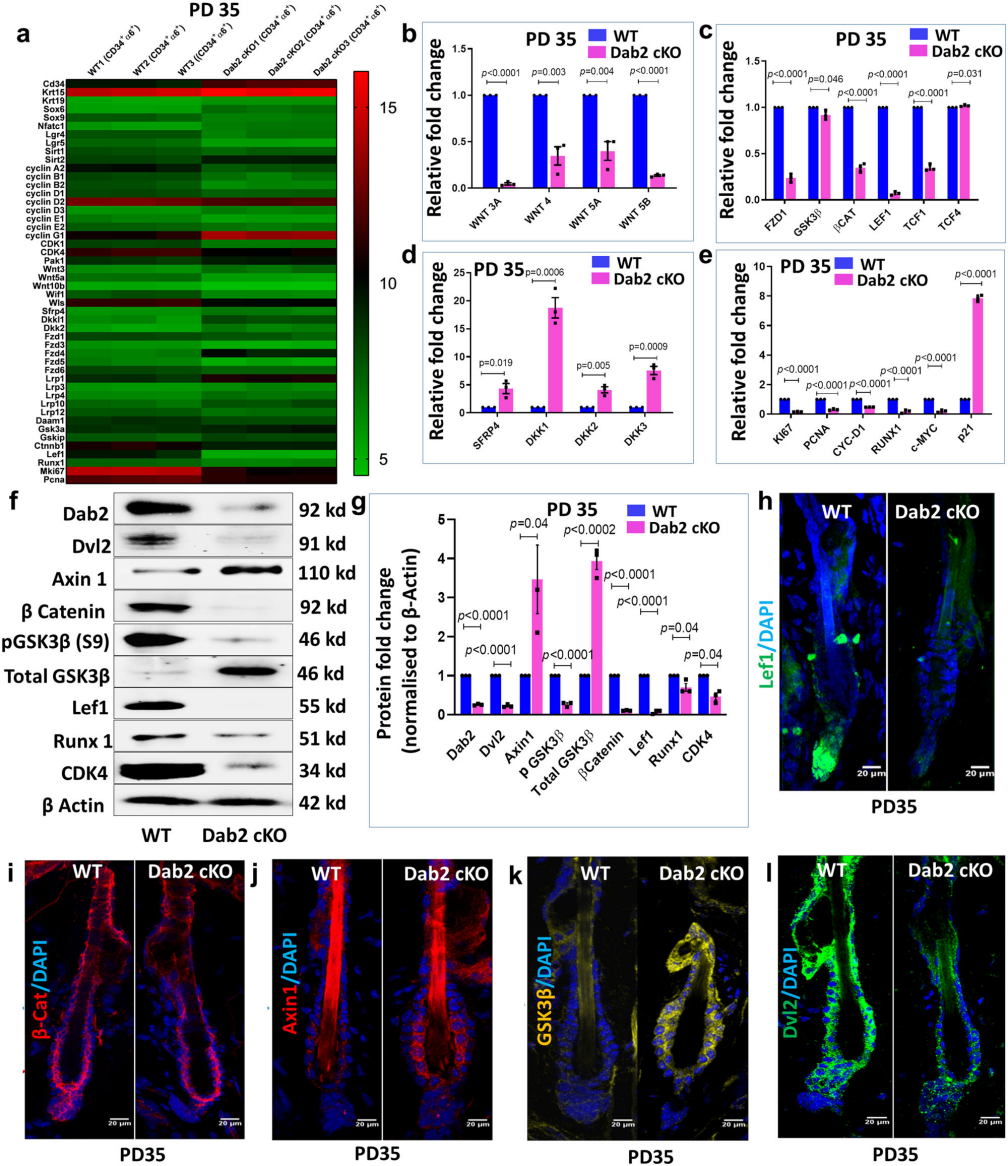
Effect of Dab2 loss on canonical Wnt signaling. **a** Heatmap showing differentially expressed genes (DEGs) related to stemness, cell-cycle, and Wnt signaling in FACS sorted WT and Dab2-cKO HFSCs at PD35, *n* = 3 independent biological replicates for each genotype. **b** qPCR analysis of Wnt ligands expression in FACS sorted WT and Dab2-cKO HFSCs at PD35, *n* = 3 independent biological replicates for each genotype. **c** qPCR analysis of Wnt signaling genes expression in FACS sorted WT and Dab2-cKO HFSCs at PD35, *n* = 3 independent biological replicates for each genotype. **d** qPCR analysis of Wnt inhibitors expression in FACS sorted WT and Dab2-cKO HFSCs at PD35, *n* = 3 independent biological replicates for each genotype. **e** qPCR analysis of Wnt target genes expression in FACS sorted WT and Dab2-cKO HFSCs at PD35, *n* = 3 independent biological replicates for each genotype. **f** Immunoblot analysis of indicated proteins in WT and Dab2-cKO whole skin tissue at PD35, *n* = 3 independent biological replicates for each genotype. **g** Graph showing relative fold change of indicated proteins in WT and Dab2-cKO whole skin tissue at PD35. **h** IFA of Lef1 expression in WT and Dab2-cKO cryopreserved skin sections at PD35, *n* = 3 independent biological replicates for each genotype. **i** IFA of β-catenin expression in WT and Dab2-cKO cryopreserved skin sections at PD35, *n* = 3 independent biological replicates for each genotype. **j** IFA of Axin1 expression in WT and Dab2-cKO cryopreserved skin sections at PD35, *n* = 3 independent biological replicates for each genotype. **k** IFA of Total GSK3β expression in WT and Dab2-cKO cryopreserved skin sections at PD35, *n* = 3 independent biological replicates for each genotype. **l** IFA of Dvl2 expression in WT and Dab2-cKO cryopreserved skin sections at PD35, *n* = 3 independent biological replicates for each genotype. (Data represent mean ± SEM, *p < 0.05, **p < 0.01, ***p < 0.001, ****p < 0.0001 obtained by students *t*-test).

To understand the Dab2-mediated molecular mechanisms involved in the activation of HFSCs, we performed expression profiling on FACS sorted HFSCs of WT and Dab2-cKO mice at PD35. We observed a significant decrease in the expression of Wnt pathway genes such as β-catenin, Lef-1, Runx1 and cell-cycle related genes such as cyclins (A2, B1, B2, E1, E2, D1, D2), cyclin-dependent kinases (CDK1, CDK4), Ki67, and PCNA in Dab2-cKO HFSCs (Fig. 7a and Supplementary Fig. 7a). The results were further validated by qPCR analysis (Fig. 7b–e). Western blot analysis of full-thickness skin lysates at PD35 revealed significantly reduced levels of Dvl2, β-catenin and Lef1 and elevated levels of Axin1 and Total GSK3β in Dab2-cKO (Fig. 7f, g and Supplementary Fig. 11). The Wnt/β-catenin targets such as CDK4 and Runx1 was also significantly reduced in Dab2-cKO. The stabilized β-catenin expression in the HG and bulge region is a sign of telogen-anagen transition^27^. Nuclear Lef1 was observed in the HG region of WT but was completely absent in Dab2-cKO HGs (Fig. 7h) and reduced level of β-catenin was observed in the lower bulge and HG region of Dab2-cKO (Fig. 7i). We also observed an increased expression of Axin (Fig. 7j) and Total GSK3β in Dab2-cKO bulge (Fig. 7k). Dvl2, which is known to regulate the stability of Axin, was also reduced in Dab2-cKO bulge (Fig. 7l). Together, these data indicate that Dab2 loss results in the inactivation of the canonical Wnt signaling which in turn is responsible for the delayed activation and reduced proliferation of HFSCs at PD35.

### Expression profiling at the second telogen revealed inactivation of the canonical Wnt pathway in Dab2-cKO HFSCs

**Fig. 8:**
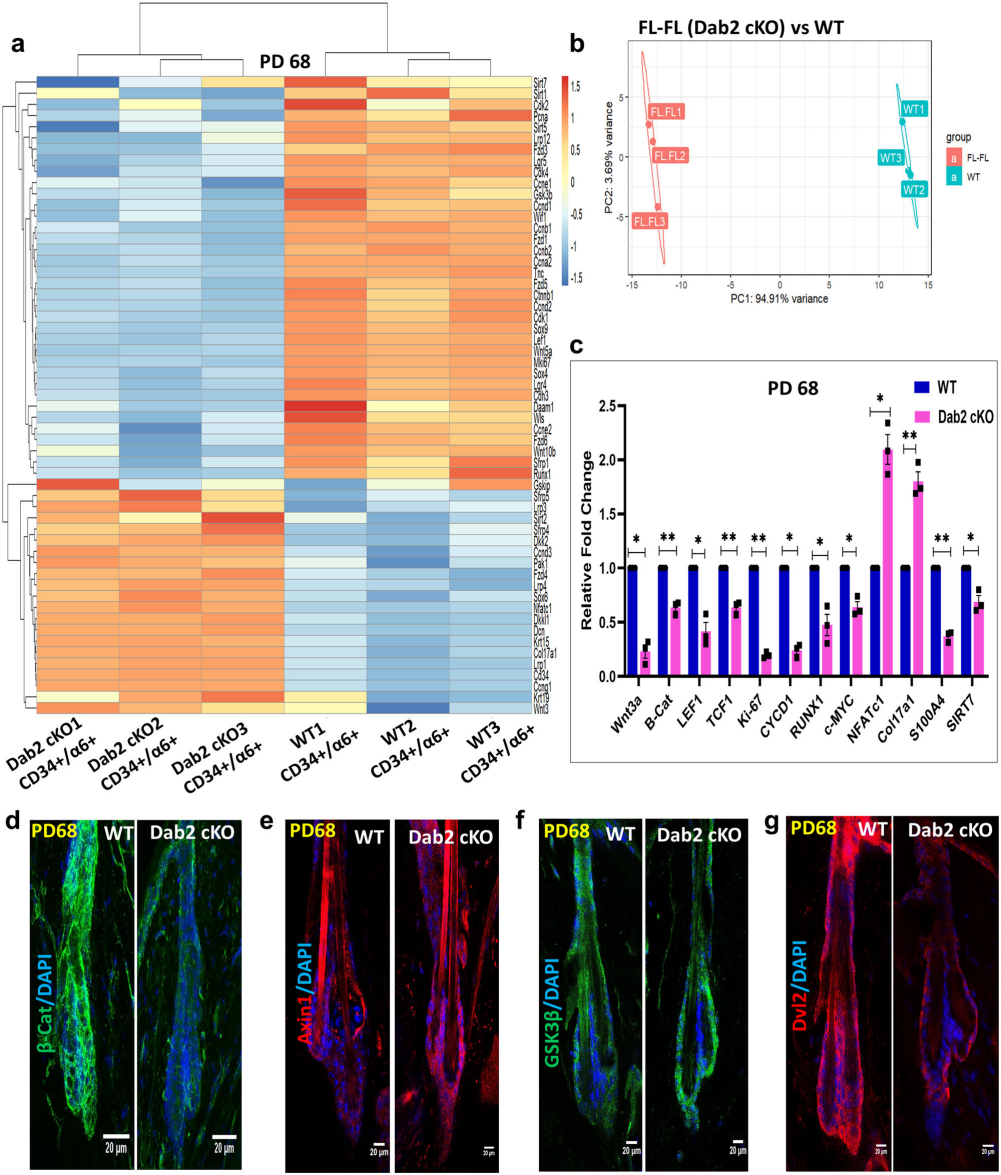
Expressing profiling of HFSCs at PD68 (second telogen). **a** Heatmap showing DEGs related to stemness, cell-cycle, and Wnt signaling in FACS sorted WT and Dab2-cKO HFSCs at PD68, *n* = 3 independent biological replicates for each genotype. **b** Principal component analysis (PCA) plot of RNA-seq data for three independent biological replicates corresponding to the samples from WT (green boxes) and Dab2-cKO (red boxes). **c** qPCR analysis of indicated genes in FACS sorted WT and Dab2-cKO HFSCs at PD68, *n* = 3 independent biological replicates for each genotype. **d** IFA of β- Catenin in WT and Dab2-cKO mice at PD68, *n* = 3 independent biological replicates for each genotype. **e** IFA of Axin1 in WT and Dab2-cKO mice at PD68, *n* = 3 independent biological replicates for each genotype. **f** IFA of Total GSK3-β in WT and Dab2-cKO mice at PD68, *n* = 3 independent biological replicates for each genotype. **g** IFA of Dvl2 in WT and Dab2-cKO mice at PD68, *n* = 3 independent biological replicates for each genotype. (Data represent mean ± SEM, *p < 0.05, **p < 0.01, ***p < 0.001, ****p < 0.0001 obtained by students *t*-test).

The alterations observed in canonical Wnt signaling between WT and Dab2-cKO mice at PD35 could be due to a difference in the phases of the HF cycle. Therefore, we performed RNA sequencing of WT and Dab2-cKO HFSCs at PD68, when both WT and Dab cKO were at the second telogen phase (Fig. 8a, b and Supplementary Fig. 8a, b). The Wnt pathway analysis showed a significant decrease in the expression of Wnts 3a, 5a, 10b, β-catenin, and Lef-1 and a significant increase in the expression of Wnt inhibitors such as Dkk1, Dkk2, and Sfrp4. Furthermore, we observed a significant decrease in the expression of cyclin D1, Runx1 and Ki67 in Dab2-cKO HFSCs (Fig. 8a). The results obtained from RNA sequencing were further validated by qPCR analysis (Fig. 8c). IFA revealed reduced levels of Dvl2 and β-catenin and increased levels of Axin and Total GSK3β in the Dab2-cKO bulge (Fig. 8d–g). The results obtained at PD68 were similar to that observed at PD35, thereby indicating that the changes observed in canonical Wnt signaling in Dab2-cKO mice is due to loss of Dab2 per se and not due to a difference in the phases of the HF cycle.

### Reversal study with Dvl2 overexpression results in partial restoration of β-catenin activity

**Fig. 9:**
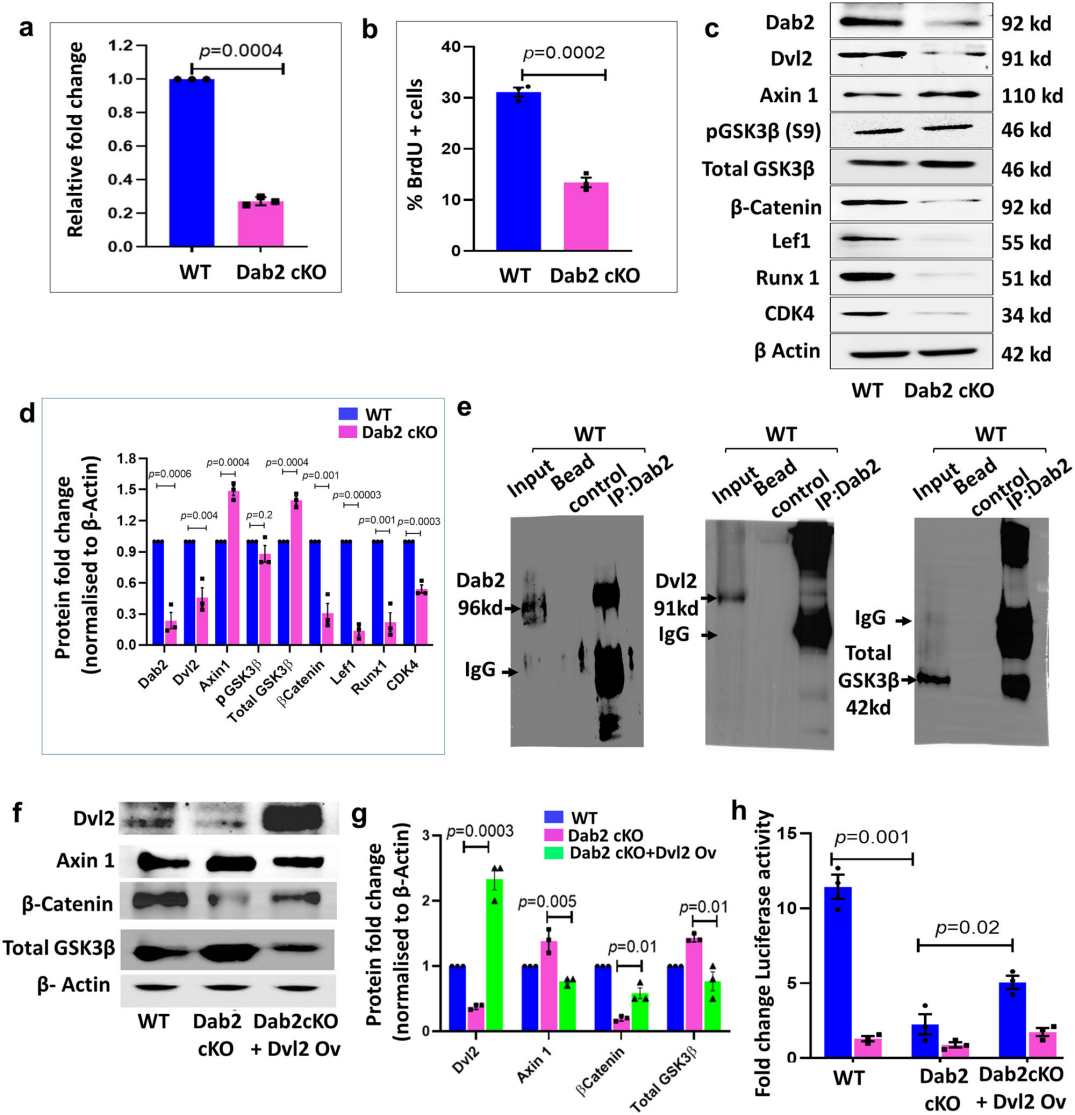
Dab2-dependent stabilization of Dvl2 is essential for activation of canonical Wnt signaling. **a** qPCR analysis of Dab2 expression in WT and Dab2-cKO primary keratinocytes, *n* = 3 independent biological replicates for each genotype. **b** Graph showing the percentage of BrdU+ cells in synchronized WT and Dab2-cKO primary keratinocytes, *n* = 3 independent experimental replicates for each genotype. **c** Immunoblot analysis of indicated proteins in WT and Dab2-cKO primary keratinocytes, *n* = 3 independent experimental replicates for each genotype. **d** Graph showing relative fold change of indicated proteins in WT and Dab2-cKO primary keratinocytes, *n* = 3 independent experimental replicates for each genotype. **e** Co-IP of Dab2 with Dab2, Dvl2, and Total GSK3β immunoblotting in WT primary keratinocytes, *n* = 3 independent experimental replicates. **f** Immunoblot analysis of indicated proteins in reversal studies using Dvl2 overexpression, *n* = 3 independent experimental replicates for each genotype. **g** Graph showing relative fold change of indicated proteins in WT, Dab2-cKO, and Dab2-cKO+Dvl2 ov primary keratinocytes, *n* = 3 independent experimental replicates. **h** Graph showing relative fold change in luciferase activity 24 h post-transfection in WT, Dab2-cKO, Dab2-cKO+Dvl2 Ov keratinocytes, *n* = 3 independent experimental replicates. (Data represent mean ± SEM, *p < 0.05, **p < 0.01, ***p < 0.001, ****p < 0.0001 obtained by students *t*-test).

To analyze the effect of Dab2 loss in vitro, we established Dab2 WT and Dab2-cKO primary keratinocytes. The 4-hydroxytamoxifen (4-OHT) treated K14CreER^-/-^ Dab2^fl/fl^ and K14CreER^+/-^Dab2^fl/fl^ primary keratinocytes were the WT control and Dab2-cKO respectively. The qPCR analysis revealed showed ~70% knockout efficiency in Dab2-cKO cells (Fig. 9a). FACS analysis revealed nearly 50% reduction in the number of BrdU+ cells in Dab2-cKO (Supplementary Fig. 9a and Fig. 9b). Similarly, the CFA revealed that Dab2-cKO cells formed significantly reduced number of colonies (Supplementary Fig. 9b, c). These results indicate that loss of Dab2 results in reduced proliferative capacity of primary keratinocytes. Next, western blot analysis revealed ~70% reduction in the expression level of β-catenin in Dab2-cKO keratinocytes. We observed a significant increase in Axin1 and Total GSK3β levels and ~50% reduction in Dvl2 levels in Dab2-cKO keratinocytes (Fig. 9c, d and Supplementary Fig. 12). Moreover, β-catenin was also downregulated at the mRNA level in Dab2-cKO keratinocytes (Supplementary Fig. 9d). Further, Wnt targets such as Lef1, Runx1, and CDK4 were significantly downregulated both at the mRNA (Supplementary Fig. 9d) and protein level in Dab2-cKO keratinocytes (Figs. 9c, d and Supplementary Fig. 12). Downregulation of Dvl2, the upstream effector of Wnt signaling led us to investigate whether Dab2 mediated its effect on canonical Wnt signaling through Dvl2. Co-immunoprecipitation (Co-IP) studies were performed using Dab2 and Dvl2 antibodies in both WT and Dab2-cKO cells. IP with anti-Dab2 antibody and immunoblotting with the same antibody in both WT and Dab2-cKO cells clearly showed the Dab2 antibody pull-down specificity and efficiency (Fig. 9e and Supplementary Figs. 10a,  13a). Furthermore, in WT cells, IP with an anti-Dab2 antibody efficiently pulled down Dvl2, which was evident from the Dvl2 level seen in the western blot using the anti-Dvl2 antibody thereby indicating a strong interaction between Dab2 and Dvl2 (Fig. 9e and Supplementary Fig. 13b). However, in Dab2-cKO cells, IP with anti-Dab2 antibody followed by immunoblotting with anti Dvl2 antibody suggested a loss of interaction between Dab2 and Dvl2 (Supplementary Figs. 10a, 13b). The rescue experiments were further performed to check whether the effect of Dab2 loss is rescued by Dvl2 overexpression in Dab2-cKO keratinocytes. The results showed that Dvl2 overexpression in Dab2-cKO keratinocytes reduced the level of Axin1 and GSK3β approximately to WT levels. Consequently, there was a threefold increase in the β- catenin levels upon Dvl2 overexpression in Dab2-cKO keratinocytes (Fig. 9f, g). Further, to demonstrate directly whether the activity of β- catenin is rescued upon Dvl2 overexpression in Dab2-cKO cells, we used the TOPFlash/FOPFlash luciferase reporter system. We observed a ~80% reduction in luciferase activity in Dab2-cKO cells transfected with the TOP Flash reporter vector. The β-catenin activity increased twofold upon Dvl2 overexpression in Dab2-cKO keratinocytes (Fig. 9h). Similarly, colony-forming capacity increased twofold upon Dvl2 overexpression in Dab2-cKO keratinocytes (Supplementary Fig. 9e, f). Thus, there was a partial restoration of β-catenin activity and proliferative capacity of Dab2-cKO keratinocytes upon Dvl2 overexpression. As stated earlier, Dab2 interacts with multiple Wnt pathway components to regulate its activation. Thus, we further performed Co-IP of Dab2 with a destruction complex component, GSK3β. In WT cells, IP with anti-Dab2 antibody pulled down Total GSK3β efficiently (Fig. 9e and Supplementary Fig. 13c), while we observed a loss of interaction between Dab2 and Total GSK3β in Dab2-cKO cells (Supplementary Figs. 10a,  13c). These results indicate that in addition to Dvl2, Dab2 may also interact with and modulate the destruction of complex components to control the activation of the canonical Wnt signaling.

## Discussion

Dab2 is an important regulator of embryonic development, however, its role in adult stem cells maintenance and regulation is still obscure. To our knowledge, this is the first study which shows the involvement of Dab2 in skin biology. We have clearly shown that Dab2 is highly expressed in the skin and is actively involved in regulating the activation of HFSCs. Genes involved in cell-cycle progression are downregulated in the quiescent HFSCs as compared to activated HFSCs^4^. During the telogen-anagen transition, HFSCs gets activated and starts expressing proliferation markers such as cyclin D1 and Ki67^27^. In our study, decreased expression of Ki67, cyclins B1, D1, and CDK4 in Dab2-cKO HFSCs implicates that Dab2 loss leads to perturbed cell-cycle progression resulting in delayed activation of HFSCs. Depilation studies showed that even external activation signals cannot reverse the inhibitory effect of Dab2 deletion on the activation potential of HFSCs. Hitherto, several reports have shown the anti-proliferation role of Dab2 in murine macrophages, ovarian cancer, breast cancer, prostrate, etc.^28–32^. However, our study shows that loss of Dab2 results in delayed activation and impaired proliferation of HFSCs.

Our results show that Dab2 plays a role in maintaining the self-renewal capacity of HFSCs. During anagen, Runx1 expression increases in the bulge, which in turn represses p21 expression to promote self-renewal. Downregulation of Runx1, CDK4, PCNA, and Ki67 and upregulation of p21 in FACS sorted Dab2-cKO HFSCs may attribute to their reduced self-renewal capacity. Further, Dab2-cKO HFSCs showed an increased expression of NFATc1, which positively regulates quiescence by repressing CDK4^33,34^. Dab2-cKO HFSCs also showed an upregulation of Col17a1, which helps in maintaining quiescence of HFSCs^35^ and downregulation of S100A4 and Sirt7 which aids in the activation of HFSCs^36,37^. Sirt7-dependent Nfatc1 deacetylation and downregulation leads to the activation of HFSCs and HF cycle initiation. Loss of Sirt7 perturbs telogen-to-anagen phase transition, thereby leading to a delay in HF cycle progression in mice^36^. Our study clearly shows downregulation of Sirt7 and upregulation of NFATc1 in Dab2-cKO mice, thereby indicating that Dab2 may regulate the levels of NFATc1 by regulating Sirt7 levels.

NFATc1 and Sirtuins are also potential anti-ageing targets. Higher NFATc1 levels imposes a higher activation threshold for hair regeneration in aged mice^5^. Sirtuins such as Sirt1, Sirt6, and Sirt7, play important roles in mediating cellular responses to various oxidative and genotoxic stress^38^. Our findings show that loss of Dab2 results in downregulation of Sirt7 and upregulation of NFATc1 in aged mice, indicating that Dab2 may regulate the expression of key anti-ageing factors. The understanding of the possible link between Dab2, sirtuins, and NFATc1 levels may provide us a new insight into the Dab2-mediated regulation of HFSCs and the ageing process. In addition to NFATc1 and sirtuins, numerous other anti-ageing factors are involved in the regulation of HFSCs. Loss of Col17a1 in the mouse skin results in the loss of self-renewal property of HFSCs and differentiation into epidermal lineage^39^. Loss of Decorin reduces K15^+^ cells, thereby resulting in age-related hair loss^40^. Reduced expression of anti-ageing factors such as Col17a1, decorin, and S100a4 in Dab2-cKO mice further strengthens the relationship between Dab2 and ageing in murine skin. In the human androgenetic alopecia model, although K15^+^ HFSCs are retained, CD200^+^ and CD34^+^ hair progenitor cells are reduced, suggesting a defect in the activation potential of the HFSCs^41^. In our study, reduced expression of K15 and CD34 in aged Dab2-cKO HFSCs suggests that loss of Dab2 not only affects the HFSCs pool, but also leads to compromised activation potential of HFSCs. Studies indicate that there is an overall decrease in Wnt signaling in aged liver, lung, skeletal muscle, and brain mouse tissues as compared to young tissues^42^. Persistent expression of Dkk1 and Sfrp4 hampers the hair cycle initiation in aged mice^43^. Our data shows inhibition of the Wnt ligands and increased expression of Dkk1 and Sfrp4 in aged Dab2-cKO mice, which may also contribute to hampered hair regeneration in aged mice.

We have also shed light on the detailed molecular mechanism by which Dab2 modulates the canonical Wnt signaling to exert its function on primary keratinocytes and HFSCs. Stabilized β- catenin in the HG and bulge region initiates the telogen-anagen transition^27^. High levels of Lef1 levels in the anagen versus telogen SCs are early signs of SC activation and commitment^27^. The expression of canonical Wnt ligands such as Wnt10a and Wnt10b also increases during anagen onset as compared to resting telogen^44,45^. Dvl2 has been shown to be specifically expressed at high levels in early anagen in HF ORS and in hair precursor cells throughout anagen. Studies in transgenic mice showed that Dvl2 mimics Wnt-3 function in the control of hair growth and structure^46^. A study showed that Baicalin treatment stimulates HF growth by enhancing the expression of Wnt3a, Wnt5a, frizzled 7, Dvl2, β-catenin, and LEF1 and decreasing the expression of GSK3β^47^. In our study, we have shown that loss of Dab2 leads to reduced levels of Dvl2, β-catenin, and Lef1, which in turn is responsible for delayed HF cycle progression and compromised hair regeneration.

Previous studies have established that Sirtuins modulate the expression of Dvl proteins, in turn regulating the canonical Wnt signaling. Sirt1 forms a complex with Dvl2 and positively regulates Dvl2^48^. Loss of Sirt1 results in reduced expression of Dvl2, β-catenin, and Wnt/Dvl target genes such as cMyc and cyclin D1, thereby leading to reduced proliferation of C2C12 myoblast cells. Sirt2-mediated deacetylation of the K68 residue of Dvl2 inhibits Dvl2 homo-polymerization, resulting in the dissociation of the Dvl2 signalosomes and downregulation of the Wnt signaling^49^. Our data shows that loss of Dab2 leads to downregulation of Dvl2 and sirtuins such as Sirt2 and Sirt7, indicating that Dab2 may affect the expression of Dvl2 by regulating the levels of sirtuin genes. Thus, the understanding of the possible link between Dab2 and sirtuins will provide a new insight into the Dab2-mediated regulation of the canonical Wnt signaling.

Our study shows that Dvl2 overexpression partially restores the Wnt reporter activity in Dab2-cKO cells. If Dab2 mediated its effect on canonical Wnt signaling only via Dvl2, then DvL2 overexpression should have completely rescued the reporter activity in Dab2-cKO cells. This indicates that in addition to Dvl2, Dab2 may also regulate other Wnt signaling components. Previous studies have shown that Dab2 interacts with multiple components of Wnt signaling to regulate its activation^20^. Similarly, our findings indicate that in addition to Dvl2, Dab2 also interacts with one of the destruction complex components, GSK3β. We also observed an increase in the Total GSK3β levels in Dab2-cKO keratinocytes. It has been shown previously that activated canonical Wnt signaling leads to sequestration of GSK3β inside membrane-bound multivesicular endosomes, reducing its cytosolic levels^50^. The observations in our study indicate that Dab2 may play a vital role in the sequestration of GSK3β in early endosomes. Loss of Dab2 may affect this process, thereby stabilizing the protein in the cytoplasm. A more comprehensive interaction study in the future will help us to understand the exact mechanism by which Dab2 regulates the stability of GSK3β.

Studies indicate that various Dvl paralogs act as a branching point between canonical and noncanonical Wnt signaling. Upon specific Wnt stimulation, Dvl either interacts with the Dvl-associated activator of morphogenesis 1 (DAAM1)^51,52^ or Tiam1^52,53^ to regulate actin polymerization and cytoskeletal remodeling^52^. Dvl plays a crucial role in the activation of phospholipase C (PLC), resulting in activation of the Wnt/Ca^2+^ signaling via Nemo-like kinase-mediated TCF4 phosphorylation^54^. This inhibits the binding of β-catenin/TCF4 complex to DNA, causing inactivation of the canonical Wnt signaling. Moreover, Dvl mediates Wnt signaling both at the cytoplasmic and nuclear levels. Dvl3 interacts with c-jun and forms a complex with β-catenin–TCF- 4 on the promoter region of c-myc, a Wnt target gene, to regulate its transcription^55^. Nuclear Dvl3 also interacts with chromatin-modifying enzymes such as KMT2D, promotes H3K4 methylation, and regulates transcription in breast cancer cells^56^. Our study shows that while Dab2 loss results in the reduced expression of Dvl2, it does not affect the expression of Dvl3 isoform (Supplementary Fig. 10b, c and Supplementary Fig. 13d). This finding suggests that Dab2 primarily regulates the expression of Dvl2 in primary keratinocytes. Moreover, if Dvl3 had a compensatory effect on the canonical Wnt signaling in the absence of Dvl2, then we would not have observed a significant reduction in β-catenin activity in Dab2-cKO keratinocytes. However, Dvl3 may regulate noncanonical Wnt signaling in Dab2-cKO keratinocytes. In addition, Dvl3 may also regulate chromatin modifications in Dab2-cKO keratinocytes. Detailed molecular studies in the future may shed light as to how Dab2 differentially regulates the expression of various Dvl paralogs and activation of canonical and noncanonical Wnt signaling in keratinocytes.

**Fig. 10:**
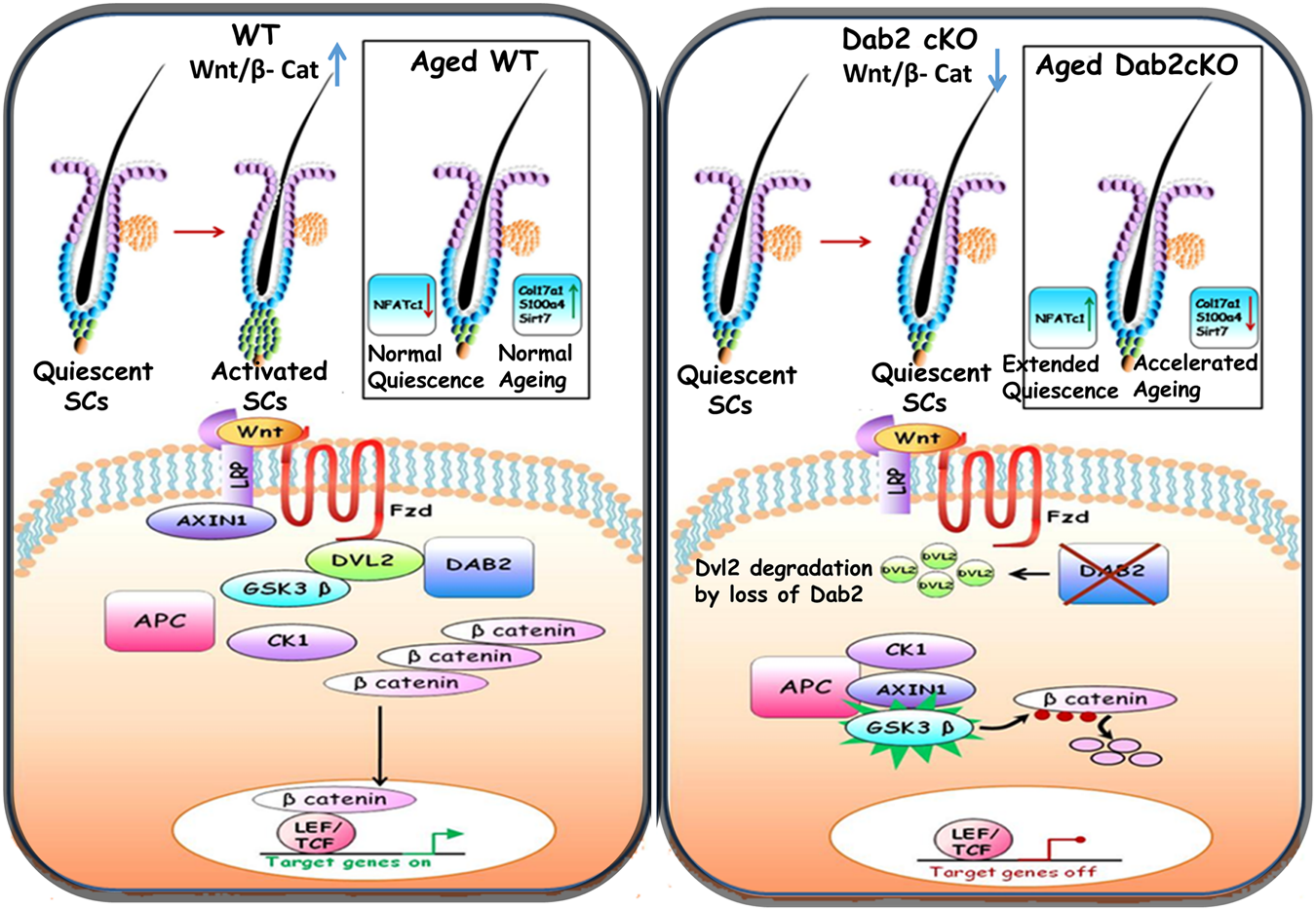
Dab2 promotes Wnt signaling and activation of HFSCs. HFSCs undergo cyclic bouts of quiescence and activation during the adult hair cycle. Quiescent HFSCs reside in a specialized niche known as the bulge (shown by blue cells). At the end of the telogen phase, HG cells (as shown by green cells at the base of the bulge) start proliferating and initiate the anagen phase. In Dab2 WT, activation of Wnt signaling leads to the activation of quiescent HFSCs. In WT follicles, Dvl2 is stabilized in the presence of Dab2. Thereafter, Axin1 is translocated to the plasma membrane and is later degraded. As a result, the β- catenin destruction complex is disrupted. Stabilized β- catenin starts accumulating in the cytoplasm, which then translocates to the nucleus. It binds to LEF/TCF proteins to regulate the expression of target genes required for activation of HFSCs. As a result, WT HFs transit from the telogen to the anagen phase. In Dab2-cKO, Dvl2 is degraded in the absence of Dab2. As a result, Axin1 remains in the cytoplasm, which prevents its degradation. As a result, the destruction complex is active, which degrades β-catenin, thereby shutting down the transcription of target genes. Therefore, in Dab2-cKO, Wnt signaling is downregulated, which prevents the activation of HFSCs. As a result, the Dab2-cKO HF remains in the telogen phase. In addition, anti-ageing factors are also downregulated in Dab2-cKO mice, which accelerates the ageing process.

Based on our present findings, we propose a model in which Dab2 plays an important role in the activation of canonical Wnt signaling. In the presence of Dab2, Dvl2 is stabilized, providing a platform for the formation of the Wnt signalosomes, which include scaffolding proteins (Axin1) and kinases (GSK3β and CK1γ)^57^. Loss of Dab2 results in the degradation of Dvl2. As a result, the destruction complex is no longer recruited to the receptor, which leads to the stabilization of the destruction complex in the cytoplasm followed by degradation of β-Catenin and inactivation of the canonical Wnt signaling (Fig. 10). In addition, Dab2 may also regulate the destruction complex directly to control the activation of Wnt signaling in HFSCs.

Thus in the present study, we have shown a context-dependent role of Dab2 in HFSCs wherein it aids in the activation of canonical Wnt signaling for HFSCs activation and HF cycle initiation. We have also shown the involvement of Dab2 in the maintenance of quiescence of HFSCs which also contributes significantly to the ageing process. These findings highlight Dab2 as a potential new target for treating ageing-related hair loss.

## Methods

### Transgenic mice

Dab2^fl/fl^ mice and K14CreER^+/−^ mice were imported from Jackson laboratories. Dab2^fl/fl^ mice were crossed with K14CreER^+/−^ to generate K14CreER^+/−^:Dab2^fl/+^ mice. Subsequent crossings were performed for eight generations to obtain two genotypes—K14CreER^-/-^Dab2^fl/fl^ (WT) and K14CreER^+/−^Dab2^fl/fl^ (Dab2-cKO). PreH2BGFP and K5Tta were procured from Jackson, USA and Prof Rune Toftgard, Sweden, respectively. Mice were maintained at the ACTREC animal house facility under specific pathogen-free conditions. All animal experiments were approved by ACTREC’s Institutional Animal Ethics Committee (IAEC). Our institutes follow CPSEA guidelines provided by India’s Ministry of Environment and Forests government for all animal experiments. We have complied with all relevant ethical regulations for animal use. IAEC project number 04/2022 was approved in April 2022.

### PCR genotyping

The tail samples of the mice were collected using sterilized scissors and forceps into a 1.5 ml Eppendorf containing 500 μl of tail lysis buffer. About 10 μl Proteinase K (Stock 20 mg/ml) was added to each tube, followed by incubation at 55–60 °C O/N. The next day, 300 μl of 5 M NaCl was added to each tube, and the tubes were shaken vigorously for 15–20 times and then placed on ice for 10 min. The tubes were then centrifuged at 7600 rpm for 10 min at 4 °C. The supernatant was collected into a fresh Eppendorf tube, 650 μl of isopropanol was added and mixed gently by inverting the tubes. The tubes were incubated at RT for 15 min followed by centrifugation at 14,000 rpm for 10 min at RT to pellet out the DNA. About 50 μl of sterile water was added to the pellet in each tube and incubated in dry bath at 50 °C for 5 min to dissolve the DNA completely. The DNA concentration and purity (260/280 ratio) was measured by Nanodrop quantification. For 25 μl PCR reaction, 1 to 2 μl of DNA was used. Refer to Supplementary Data 4 for details of the primer sequences used for genotyping.

#### Tamoxifen dosing and confirmation of knockout efficiency

To determine the dose of tamoxifen required for Cre activation, ROSA-YFP:K14CreER^+/−^ mice were injected intraperitoneally with tamoxifen (75 mg/kg dissolved in corn oil) for 5 consecutive days. After 48 h of the last injection, mice were sacrificed and checked for YFP expression by IFA in dorsal skin sections. For experimental purposes, both K14CreER^−/−^Dab2^fl/fl^ and K14CreER^+/−^Dab2^fl/fl^ (Dab2-cKO) were injected intraperitoneally with tamoxifen (75 mg/kg dissolved in corn oil) for 5 consecutive days. To check the Dab2 KO efficiency in the epidermis, mice were sacrificed at PD68, and dorsal skin sections were collected. We incubated the skin sections in 10 ml 0.25% trypsin O/N at 4 °C. The next day, 10 ml fresh trypsin was added followed by incubation at 37 °C for 30 min. The epidermis was then peeled off from the dermis using a surgical blade and further processed for single-cell suspension. The cells were lysed in trizol for RNA isolation.

### Histology and IFA

Mice dorsal skin sections were collected, fixed in 4% formalin or directly embedded, and cryopreserved in OCT compound (Tissue-Tek). Haematoxylin and Eosin staining (H&E) was performed on 5-µm thick paraffin-embedded for analysis of HF cycle phases. For the Immunofluorescence assay (IFA), the OCT-embedded sections were fixed using either acetone or 4% paraformaldehyde. These sections were then washed with 1X PBS and treated with 0.1 to 0.3% triton-X 100, followed by 5% NGS/NDS (normal goat serum and normal donkey serum) blocking at RT for 1 h. Primary antibody incubation was done O/N at 4 °C. Sections were washed with 1X 0.2% PBST, followed by fluorophore-conjugated secondary antibody incubation for 1 h at RT. The sections were then washed with 1X PBS, treated with DAPI (4′, 6′-diamidino-2-phenylindole) and mounted with antifade. Imaging was done using LSM 780 confocal microscope (Zeiss). Immunostaining details in Supplementary Data 4.

### Flow cytometry

Dorsal skin sections of WT and Dab2-cKO mice at were collected at PD35, PD68 and at 52 weeks. Excess fats were removed from the dermal side by scrapping with a scalpel blade. Skin was kept in 10 ml of 0.25% trypsin O/N at 4 °C. Next day, 10 ml of fresh 0.25% trypsin was added in all the plates containing skin and incubated at 37 °C for 30 min. The epidermal side was scraped to separate the epidermis from the dermis. Epidermal cells were separated from the dermis by constant scraping. E-media containing 15% FBS was added to neutralize the trypsin. The solution was passed through a pipette multiple times to make a single-cell suspension that was further strained using 70 µm and then 40 µm strainers. The filtered solution was centrifuged at 2000 rpm for 5 min at 4 °C. The cell pellet was washed by using ice-cold 1X PBS and centrifuged again. The cell pellet was then dissolved in 750 ul–1 ml of 5% chelated FBS in 1X PBS (FACS buffer) and stained with the antibodies of HFSCs markers (CD34 and α6-integrin) to sort HFSCs using FACS Aria machine (BD Biosciences). FACS staining and gating strategies (Supplementary Data 2,  3) and immunostaining is mentioned in Supplementary Data 4. The HFSCs were directly sorted into the E-media for colony-forming efficiency or into the RNA lysis buffer for RNA extraction.

### Proliferation dynamics study

We crossed K14CreER^+/−^Dab2fl/fl and pTRE–H2BGFP (CD1) to obtain K14CreER^+/−^Dab2^fl/fl^:pTRE–H2BGFP background. K14Cre^+/−^Dab2^fl/fl^:pTRE–H2BGFP mice were crossed with K5TtA (FVB1) mice to obtain K14Cre^+/−^Dab2f^l/fl^:pTRE–H2BGFP:K5TtAbackground. Furthermore, crosses were performed for F8 (filial) generations to obtain mice in pure Dab2^fl/fl^ background (Fig. 3h). pTRE–H2BGFP expression gets activated upon tetR–VP16 protein binding to the tetracycline response element (TRE) DNA fragment. It can be shut down by feeding the mice with doxycycline (doxy) i.e., an analog of tetracycline, for a desired time period (chase). The H2BGFP fluorescence gets diluted in cells by twofold at each division. We assessed the H2BGFP fluorescence by performing FACS using the stem cell markers (CD34 and α6-integrin). The H2BGFP fluorescence was measured after 14 and 38 days chase (Fig. 3i), which displayed distinctive peaks of fluorescence of HFSCs with peaks being labeled as Peaks 0-7; H2BGFP peak 0 representing undivided cells with highest H2BGFP intensity and GFP peak 7 representing cells with lowest H2BGFP. FACS gatings are mentioned in Supplementary Data 3, and antibodies (Supplementary Data 4).

### qPCR analysis

RNA was extracted from mouse skin and primary keratinocytes by the Trizol method. cDNA synthesis kit (Invitrogen, Carlsbad, CA) was used for cDNA synthesis. qPCR was performed using SYBR. The Ct values were normalized with respect to the expression level of β-actin Ct values in respective samples, and fold change was calculated. Refer to Supplementary Data 4 for details of the primer sequences used for qPCR analysis.

### In vivo BrdU incorporation assay

BrdU was injected intraperitoneally with BrdU (50 mg/kg of body weight) at PD30 accompanied by 0.8 mg/ml BrdU in water for three days (PD30–32). Mice were sacrificed and skin sections were collected at PD32. BrdU was exposed by treating the tissue sections with 2 N HCL for 1 h at 37 °C and detected by the anti-BrdU antibody through IFA. (Supplementary Data 4).

### In vitro BrdU proliferation assay by FACS

To perform the BrdU proliferation assay in in vitro, cells were synchronized by serum starvation for 24 h. Further, we have used the 10 µM final BrdU concentration to label the cells for 24 h. Further, 1 × 10^6^ cells were harvested, followed by fixation and permeabilization by BD Cytofix/Cytoperm buffer for 30 min. on ice. Cells were washed by using the 1X wash buffer. Cells were treated with DNase I for 1 h at 37 °C to expose the incorporated BrdU. The BrdU staining was carried out by using the anti-BrdU FITC antibody for 20 min at RT. (Supplementary Data 4). Finally, cells were resuspended in the 250 µl staining buffer, and stained cells were acquired on a flow cytometer (Attune NXT, Thermo Fisher).

### In vivo BrdU label retention assay

Pups were injected with BrdU at the dose of 50 mg/kg body weight for every 12 h from PD3–5. The pups injected with BrdU were subjected to tamoxifen treatment from PD22–26 to generate knockout at PD26 and were chased upto PD68. Skin tissues were collected at PD68 fixed in 4% PFA, treated with 2 N HCL for 1 h at 37 °C followed by primary and secondary antibody incubation for determination of LRCs^58^. (Supplementary Data 4).

### Microarray analysis

RNA extraction from FACS-purified HFSCs was done using the absolute RNA miniprep kit as described in the manufacturer’s procedure (Agilent Technologies). The RNA quality was assessed by the Agilent RNA 6000 Pico kit on the Agilent 2100 bioanalyzer. For microarray analysis, 1 ng RNA was amplified by using the Gene Chip® WT Pico amplification Kit (Affymetrix, USA) as per the manufacturer’s instructions. Differentially expressed genes were analysed using Transcriptome Analysis Console (TAC) software.

### RNA sequencing

The cDNA libraries at a length of 300 bp were prepared from 5 ng of extracted total RNA using the SMARTer Stranded Total RNA-seq kit v3- pico input mammalian (Cat. No. 634485) from Takara Bio Inc. following instructions from the manufacturer. Total RNA was sequenced at a read depth of 150 bp (paired-end) using the Illumina NovaSeq 6000 platform. The quality of generated data was tested using FastQC version 0.11.9. The pre-processing of the Raw FASTQ files was carried out using fastp version 0.23.4. The alignment of reads was carried out against a mouse genome assembly (GrCm39) obtained from Ensembl using HISAT2 version 2.2.1. The SAM to BAM conversion and sorting (by read name) of the HISAT2 output files was carried out using SAM tools version 1.6. The quality of the alignment was determined using Qualimap version 2.2.2. The mapped reads were counted using feature Counts version 2.0.6. Differentially expressed genes (DEGs) were identified from the count data using the DESeq2 package version 1.42.0. The PCA of the DEseq2 output was plotted using the pca Explorer package version 2.28.0. The volcano plot of the DESeq2 output was plotted using the Enhanced Volcano package version 1.20.0. The heatmaps for the DEGs with *p* adj >0.05 were generated using the Complex Heatmap package version 2.18.0. The Heatmap of genes commonly associated with HFSCs regulation was generated using the pheatmap package version 1.0.12.

### Protein isolation and western blotting

Mice were sacrificed at PD35, and full-thickness dorsal skin was collected. Equal amounts of dorsal skin samples were taken for WT and Dab2-cKO and were ground to a fine powder using a liquid nitrogen mortar. The powder was dissolved in RIPA lysis buffer (Sigma, Catalog number: R0278) containing a protease inhibitor cocktail (Merck, Cat No. 04693132001). The lysate was further sonicated thrice at a frequency of 20 Hz for 10 s each. After sonication, the tubes were placed in a rocker for 30 min at 4 °C. For western blotting of primary keratinocytes, the keratinocytes were cultured in 60 mm plates. On reaching 80% confluency, the cells were washed thrice with chilled 1X PBS, followed by the addition of 100 μl RIPA lysis buffer containing protease inhibitor cocktail. Cells were scraped using a plastic scraper and lysate was collected in a 1.5 ml centrifuge tube. In both cases, the lysate was centrifuged at 12,000 × *g* for 30 min at 4 °C. Pellet was discarded and the supernatant was collected in a fresh 1.5 ml tube. The protein concentration was determined using the Pierce™ BCA Protein Assay Kit. SDS PAGE was performed and blots were developed using the Biorad Chemidoc system. Refer to Supplementary Data 4 for details of the antibodies used for immunoblotting.

### Co-IP assays

Primary keratinocytes were suspended in IP lysis buffer containing 150 mM NaCl, 25 mM Tris-HCl (pH 7.9), 5 mM MgCl_2_, 10% glycerol, 0.2 mM EDTA, 0.1% NP- 40, and protease inhibitors. The supernatant was incubated with antibodies or control IgGs O/N under rotation. About 40 ul Protein A/G agarose beads (Invitrogen, USA) were then added to the previously incubated samples at 4 °C for 4 h under rotation. The immunoprecipitates were washed in 50 uM Tris wash buffer, eluted in Laemmli loading buffer, and analyzed by immunoblotting. Immunoprecipitation and Immunostaining details in Supplementary Data 4.

### Primary keratinocyte culture

PD2 pups were sacrificed and skin was incubated in dispase (5 U/ml) overnight, followed by separation of the epidermis from the dermis. Single-cell suspension was made and centrifuged at 2000 rpm/5 min/4 ˚C. The pellet was resuspended in 1 ml calcium chelated E- media containing 15% FBS and plated onto irradiated J2-3T3 (mouse embryonic fibroblast) feeder layers. The irradiated J2-3T3 feeder cells were preconditioned in E-media. The keratinocyte cultures were co-cultured with fibroblast for eight passages, after which they started growing independently. Feeder-independent primary keratinocytes were cultured in E-media for experimental purpose^59^.

### Generation of in vitro Dab2-cKO keratinocytes

Both K14CreER^−/−^ Dab2^fl/fl^ (WT) and K14CreER^+/−^ Dab2^fl/fl^ (Dab2-cKO) primary keratinocytes were treated with 4-OHT (1 µM) for 5 consecutive days. The E-media containing 1 µM 4-OHT was replaced every 48 h till 5 days for generation of cKO keratinocytes. After 5 days, the keratinocytes were cultured and passaged in normal E-media.

### Transfection and overexpression studies

Primary keratinocytes were cultured in E-media containing 15% FBS. Transfection was carried out using Lipofectamine LTX according to the manufacturers' protocol. Cells were harvested for western blot analysis after 48 h of transfection.

### Colony-forming assay (CFA)

FACS sorted 5000 HFSCs of both WT and Dab2-cKO were cultured in vitro for 21 days. For primary keratinocytes, 10,000 cells of each genotype were plated into 35 mm plates and the plates were kept in a CO_2_ incubator at 37 °C. The colonies were allowed to grow for 14–21 days. Once the colonies are formed, they were fixed with 4% PFA. Crystal violet staining was performed to stain the colonies, and the images were captured. For CFA in transiently transfected cells, cells were cultured in puromycin selection.

### Statistics and reproducibility

Statistical analysis was done in minimum *n* = 3 independent biological replicates using unpaired student’s *t*-test with Graph Pad Prism 8. Error bar indicates either mean ± SD or mean ± SEM: ns non-significant, *p < 0.05, **p < 0.01, ***p < 0.001.

### Reporting summary

Further information on research design is available in the Nature Portfolio Reporting Summary linked to this article.

### Supplementary information

Supplementary information

Description of Additional Supplementary Files

Supplementary Data 1-4

Reporting Summary

## Data Availability

The expression profile data of HFSCs of WT and Dab2-cKO at PD35 have been submitted to the GEO database bearing the accession number GSE250300. The RNA sequencing data of HFSCs of WT and Dab2-cKO at PD68 have been submitted to the GEO database bearing the accession number GSE254311. The uncropped western blot images are available in the Supplementary Figs. 11–13. The source data for all the graphs are available in the Supplementary Data 1. FACS staining strategies are available in Supplementary Data 2. FACS gating strategies are available in Supplementary Data 3. The details of the primer sequences and the antibodies are available in the Supplementary Data 4.
