# Supplementary material for: Dab2 (Disabled-2), an adaptor protein, regulates self-renewal of hair follicle stem cells
Source: Commun Biol. 2024 May 3;7:525. doi: 10.1038/s42003-024-06047-2 (PMC11068889; doi:10.1038/s42003-024-06047-2)
Supplement: Supplementary file 1 — Supplementary information [file 42003_2024_6047_MOESM1_ESM.pdf]

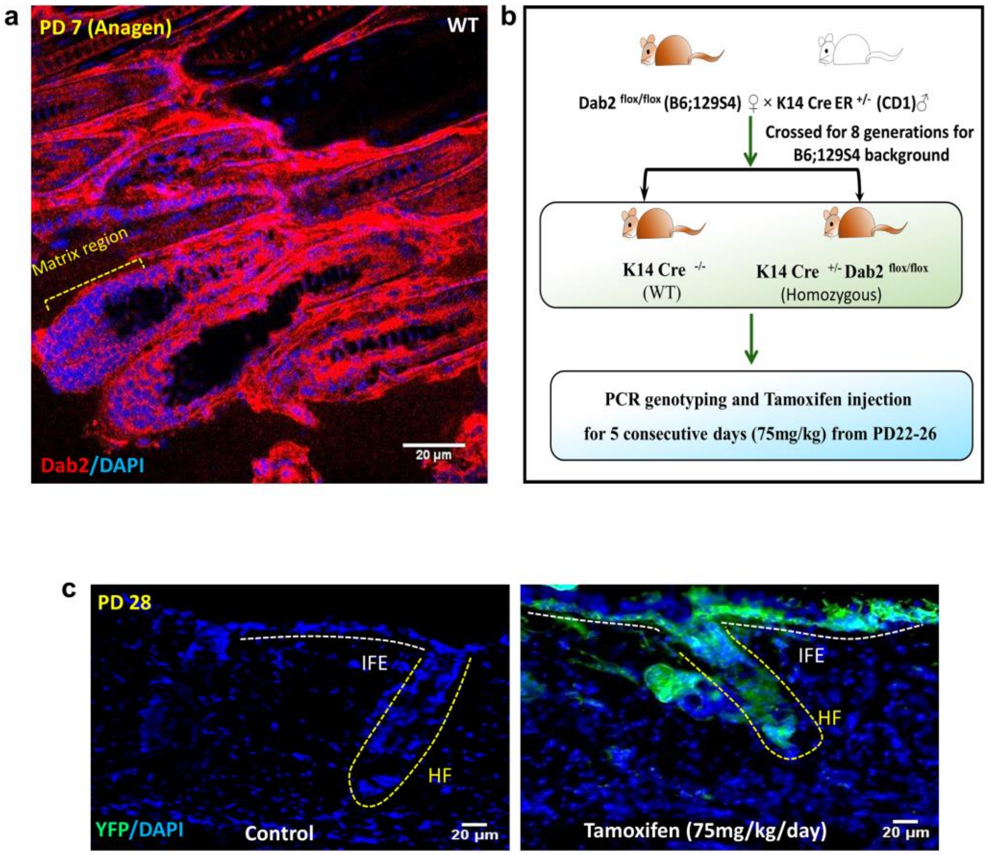

Supplementary Figure 1: Strategy and optimization of tamoxifen doses for induction of Cre activity

a) IFA showing Dab2 expression in the matrix region in WT HFs at PD7 (mid-anagen phase), n=3 independent biological replicates**.**

b) Schematic diagram showing crosses to obtain K14CreER$^{+/-}$ in Dab2$^{fl/fl}$ background and strategy for tamoxifen injection.

c) IFA showing YFP expression in control and tamoxifen injected ROSAYFP:K14CreER$^{+/-}$ mice at PD 28, n=3 independent biological replicates.

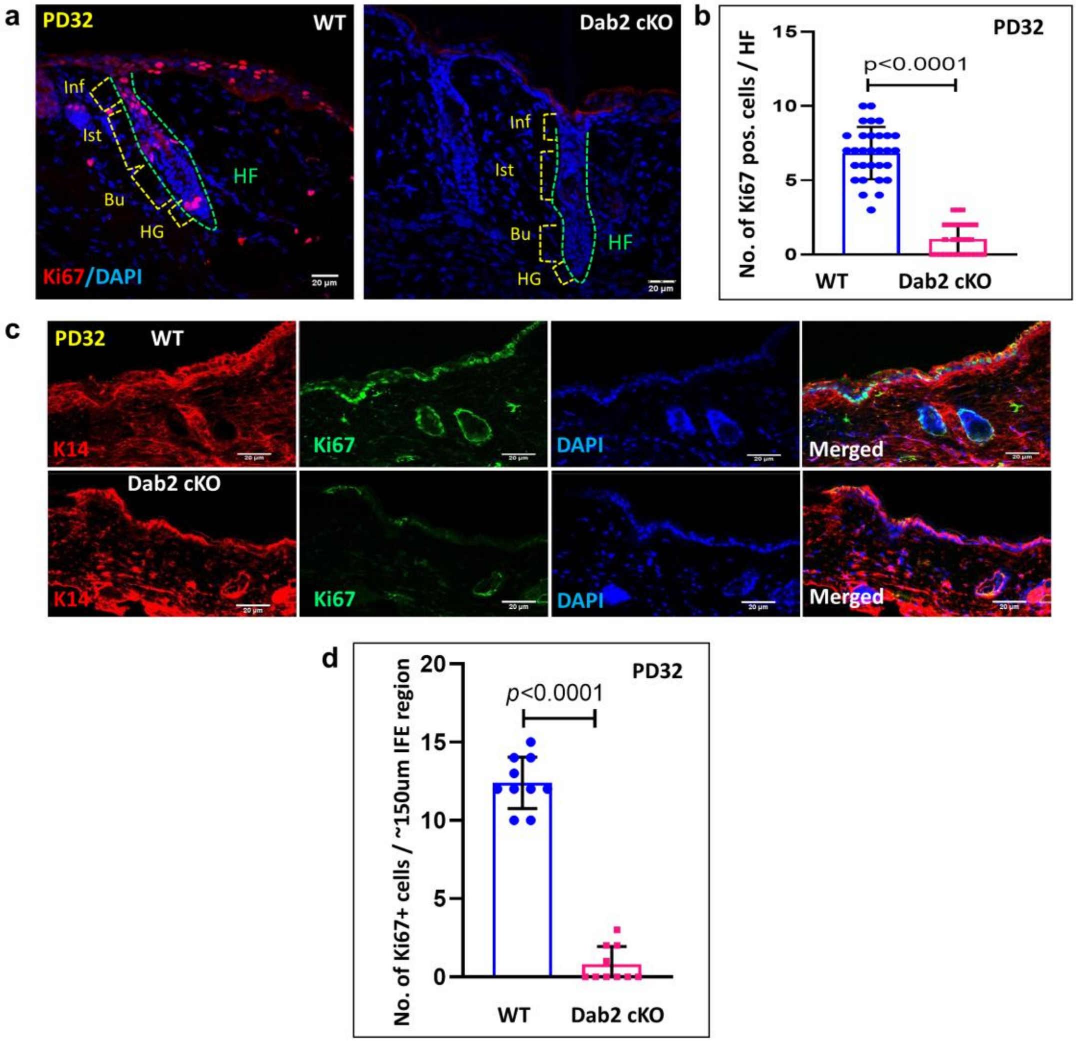

Supplementary Figure 2: Effect of Dab2 loss on the expression of Ki67

a) IFA showing Ki67+ cells/HFs at PD32 in WT and Dab2-cKO mice, n =3 independent replicates for each genotype.

b) Graph representing Ki67+ cells in HF at PD32 in WT and Dab2-cKO mice, n = 3 independent replicates for each genotype.

c) IFA showing K14 and Ki67 co-staining in the IFE region at PD32 in WT and Dab2-cKO mice, n =3 independent replicates for each genotype.

d) Graph representing Ki67+ cells in K14 expressing IFE region at PD32 in WT and Dab2-cKO mice, ~150um region of IFE per field was taken into consideration for counting Ki67 + cells, n =3 independent replicates for each genotype.

(Data represent mean ± SD, * = $p<0.05$, ** = $p<0.01$, *** = $p<0.001$, **** = $p<0.0001$ obtained by students t-test).

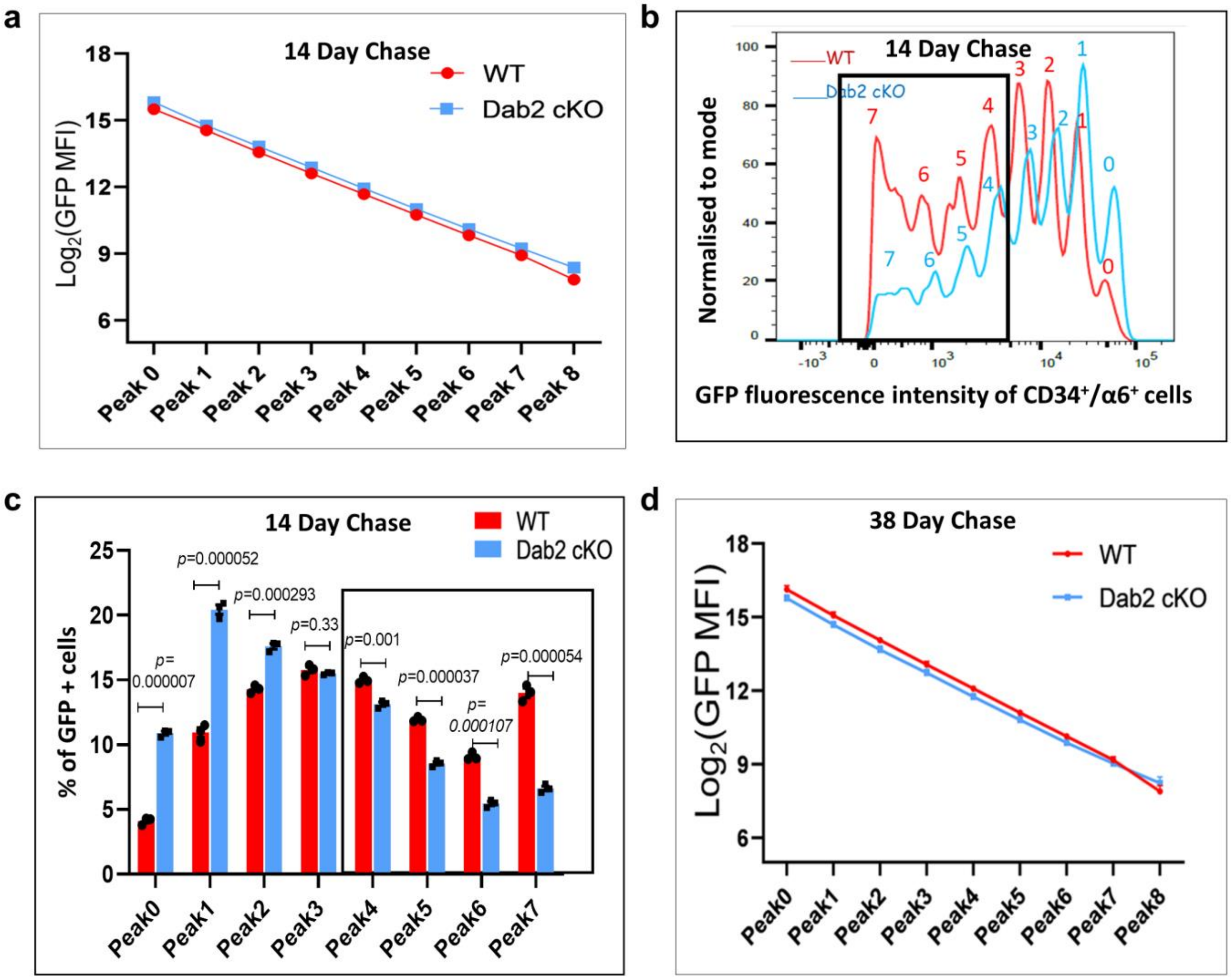

Supplementary Figure 3: Effect of Dab2 loss on the proliferation dynamics of HFSCs

a) Graph showing proper 2 fold dilution of GFP intensities with each division for 14 days chase in WT and Dab2-cKO mice, n = 3 independent biological replicates for each genotype.

b) GFP intensity peaks showing H2B-GFP dilution of FACS sorted WT and Dab2-cKO HFSCs after Doxycycline chase from PD30 to PD44, n = 3 independent biological replicates for each genotype.

c) Graph showing percentage of GFP+ cells in each GFP intensity peaks. Peak0 represents cell fractions with highest GFP intensities, Peak7 represents cell fractions with lowest GFP intensities, n = 3 independent biological replicates for each genotype.

d) Graph showing proper 2 fold dilution of GFP intensities with each division for 38 days chase in WT and Dab2-cKO mice, n = 3 independent biological replicates for each genotype.

(Data represent mean ± SEM, * = $p<0.05$, ** = $p<0.01$, *** = $p<0.001$, **** = $p<0.0001$ obtained by students t-test).

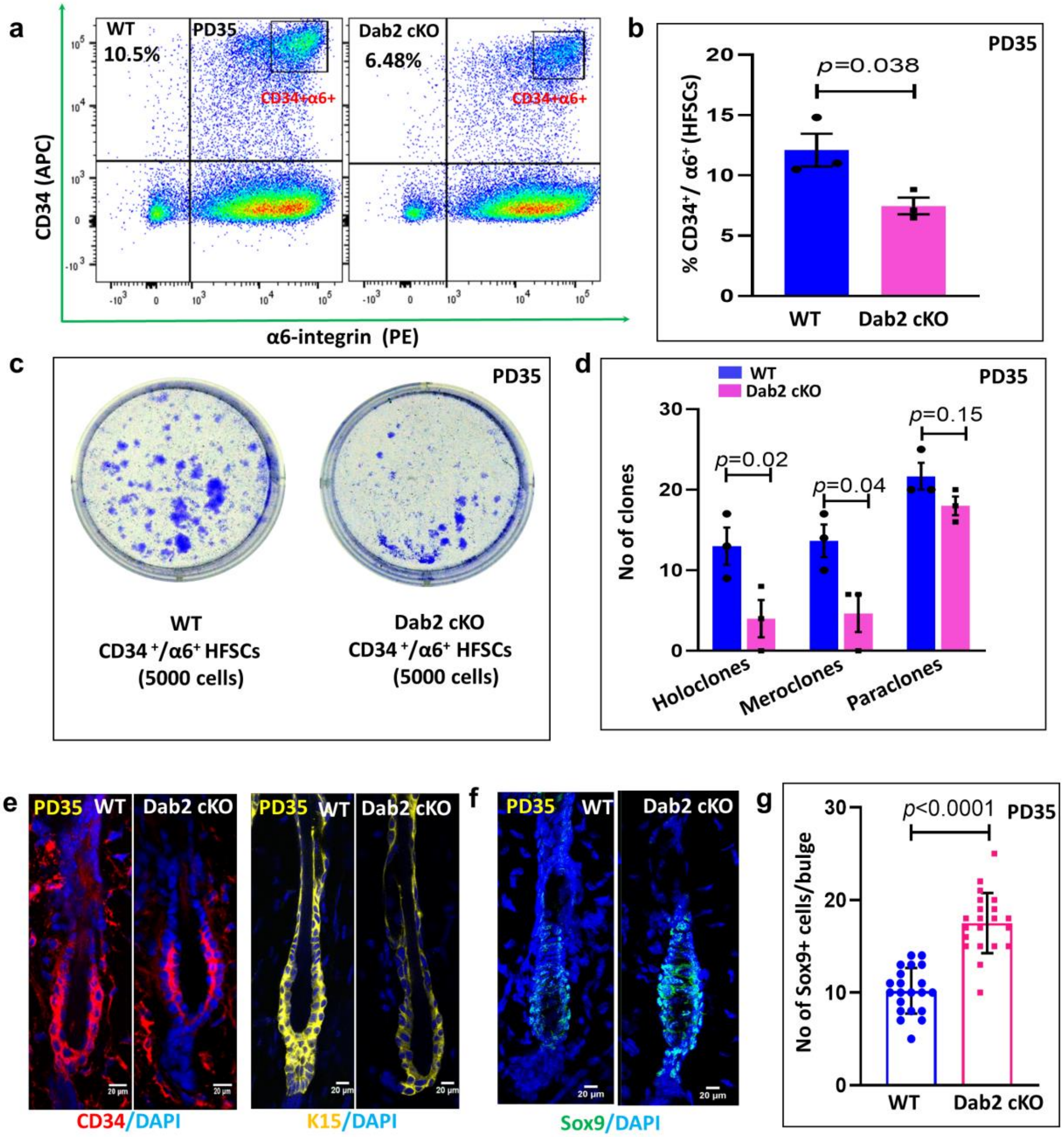

Supplementary Figure 4: Effect of Dab2 loss on the HFSCs population at PD35

a) FACS analysis showing CD34+/α6+ HFSCs population at PD35 in WT and Dab2-cKO mice, n=3 independent biological replicates for each genotype.

b) Graph showing percentage of CD34+/α6+ HFSCs population at PD35 in WT and Dab2-cKO mice, n=3 independent biological replicates for each genotype, mean ± SEM , * = p<0.05, ** = p<0.01, *** = p<0.001, **** = p<0.0001 obtained by students t-test.

c) Colonies formed by FACS sorted WT and Dab2-cKO HFSCs at PD35 when allowed to grow for 3 weeks.

d) Graph showing no. of holoclones, meroclones and paraclones formed by FACS sorted WT and Dab2-cKO HFSCs at PD35, n=3 independent replicates for each genotype, mean ± SEM, * = $p<0.05$, ** = $p<0.01$, *** = $p<0.001$, **** = $p<0.0001$ obtained by students t-test.

e) IFA of CD34 and K15 expression in WT and Dab2-cKO mice at PD35, n=3 independent biological replicates for each genotype.

f) IFA of Sox9 expression in WT and Dab2-cKO mice at PD35, n=3 independent biological replicates for each genotype.

g) Graph showing Sox9+ cells in WT and Dab2-cKO mice at PD35, n=3 independent biological replicates for each genotype, mean ± SD, * = $p<0.05$, ** = $p<0.01$, *** = $p<0.001$, **** = $p<0.0001$ obtained by students t-test.

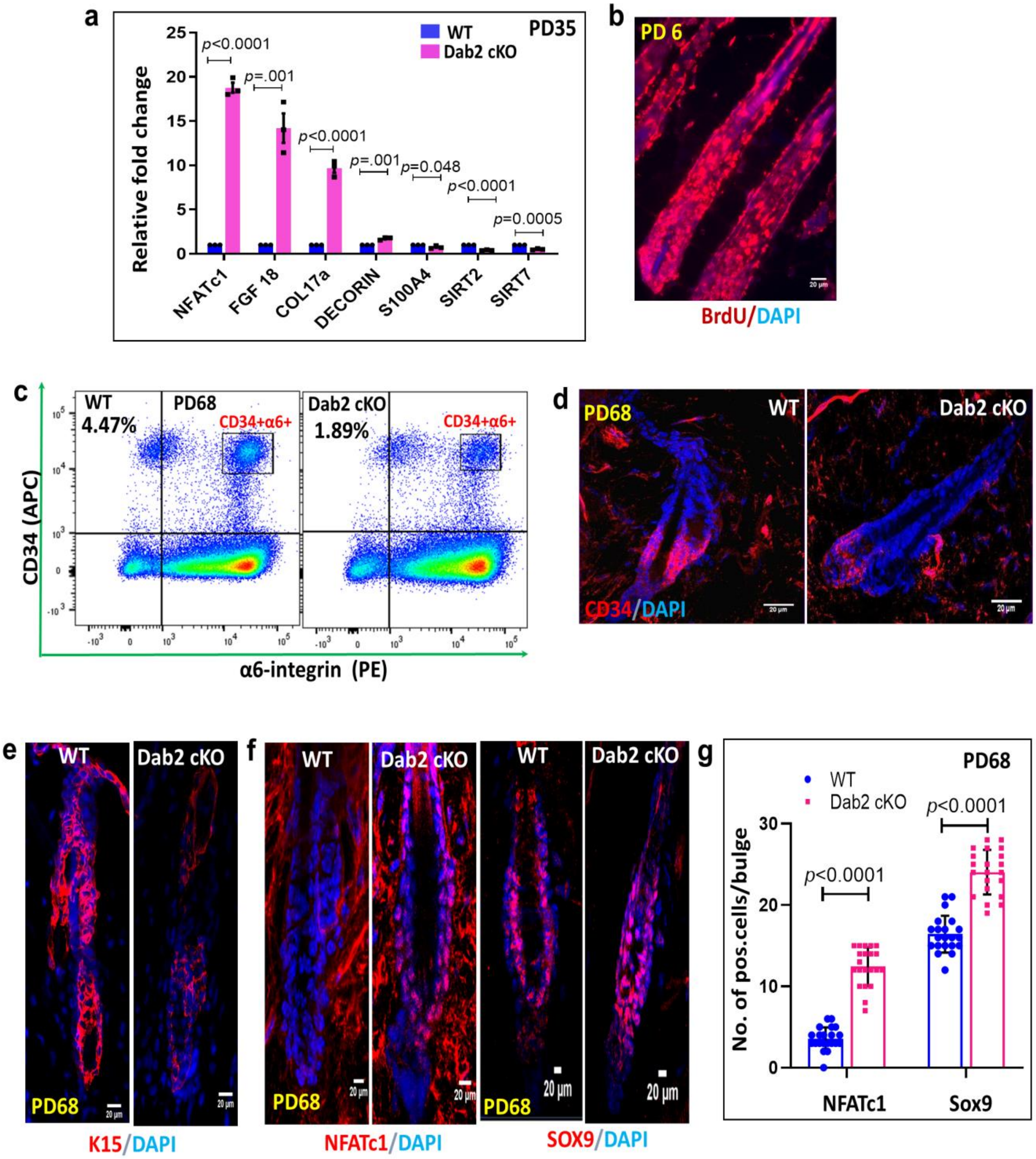

Supplementary Figure 5: Effect of Dab2 loss on the HFSCs population at PD68 (2nd telogen)

a) qPCR analysis of niche related genes expression in WT and Dab2-cKO HFSCs at PD35, n=3 independent biological replicates for each genotype, mean ± SEM, * = p<0.05, ** = p<0.01, *** = p<0.001, **** = p<0.0001 obtained by students t-test.

b) IFA of BrdU expression at PD6 after BrdU injection in neonatal pups from PD3-PD5 showing efficient BrdU labelling.

c) FACS analysis showing CD34+/α6+ HFSCs population at PD68 in WT and Dab2-cKO mice, n=3 independent biological replicates for each genotype.

d) IFA of CD34 expression in WT and Dab2-cKO mice at PD68, n=3 independent biological replicates for each genotype.

e) IFA of K15 expression in WT and Dab2-cKO mice at PD68, n=3 independent biological replicates for each genotype.

f) IFA of Sox9, NFATc1 expression in WT and Dab2-cKO mice at PD68, n=3 independent biological replicates for each genotype.

g) Graph showing NFATc1 and Sox9+ cells in WT and Dab2-cKO mice at PD68, n=3 independent biological replicates for each genotype, mean ± SD, * = $p<0.05$, ** = $p<0.01$, *** = $p<0.001$, **** = $p<0.0001$ obtained by students t-test.

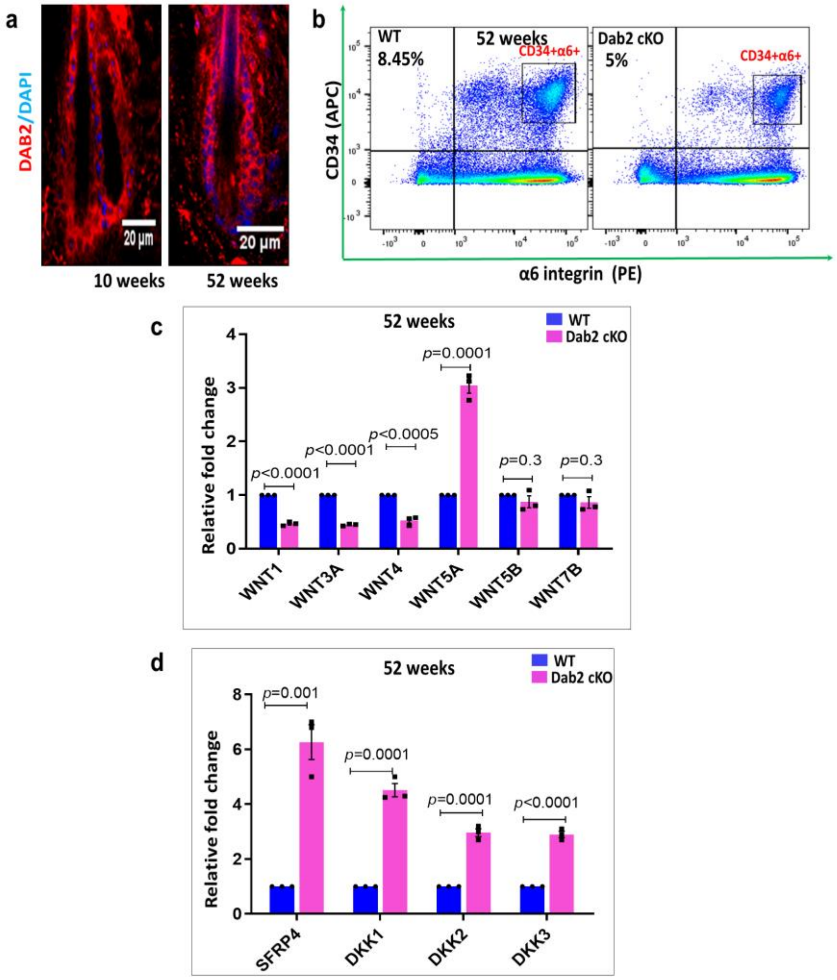

Supplementary Figure 6: Effect of Dab2 loss on the HFSCs population in aged (52 weeks old) mice

a) IFA of Dab2 expression in 10 weeks & 52 weeks old WT and Dab2-cKO mice, n=3 independent biological replicates for each genotype.

b) FACS analysis showing CD34+/α6+ HFSCs population at 52 weeks in WT and Dab2-cKO mice, n=3 independent biological replicates for each genotype.

c) qPCR analysis of Wnt ligands expression in 52 weeks old WT and Dab2-cKO skin, n=3 independent biological replicates for each genotype

d) qPCR analysis of Wnt inhibitors expression in 52 weeks old WT and Dab2-cKO skin, n=3 independent biological replicates for each genotype.

(Data represent mean ± SEM, * = $p<0.05$, ** = $p<0.01$, *** = $p<0.001$, **** = $p<0.0001$ obtained by students t-test).

a

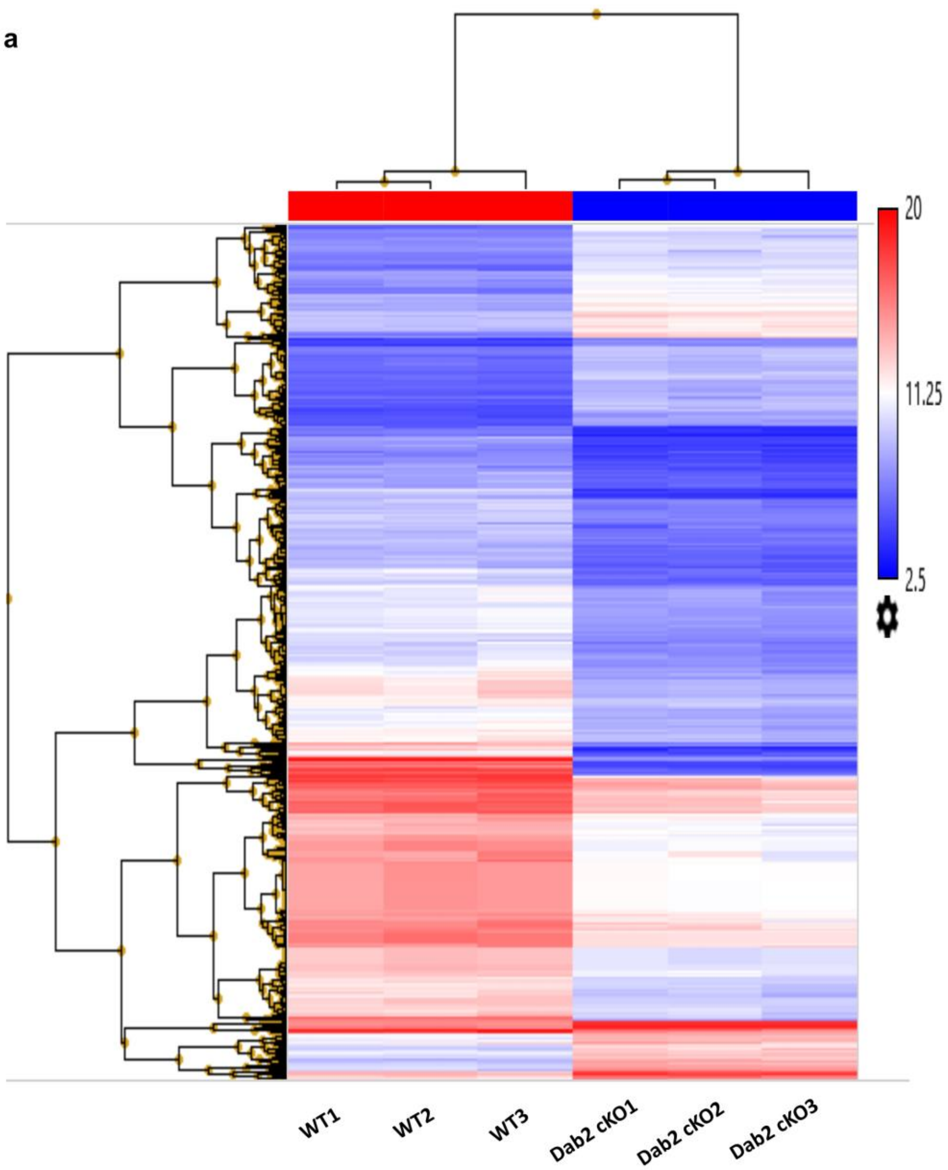

Supplementary Figure 7: Dab2 loss results in differential expression of genes at PD35

a) Heatmap showing DEGs in FACS sorted WT and Dab2-cKO HFSCs at PD35, n=3 independent biological replicates. The analysis was performed using Transcriptome Analysis Console (TAC) software.

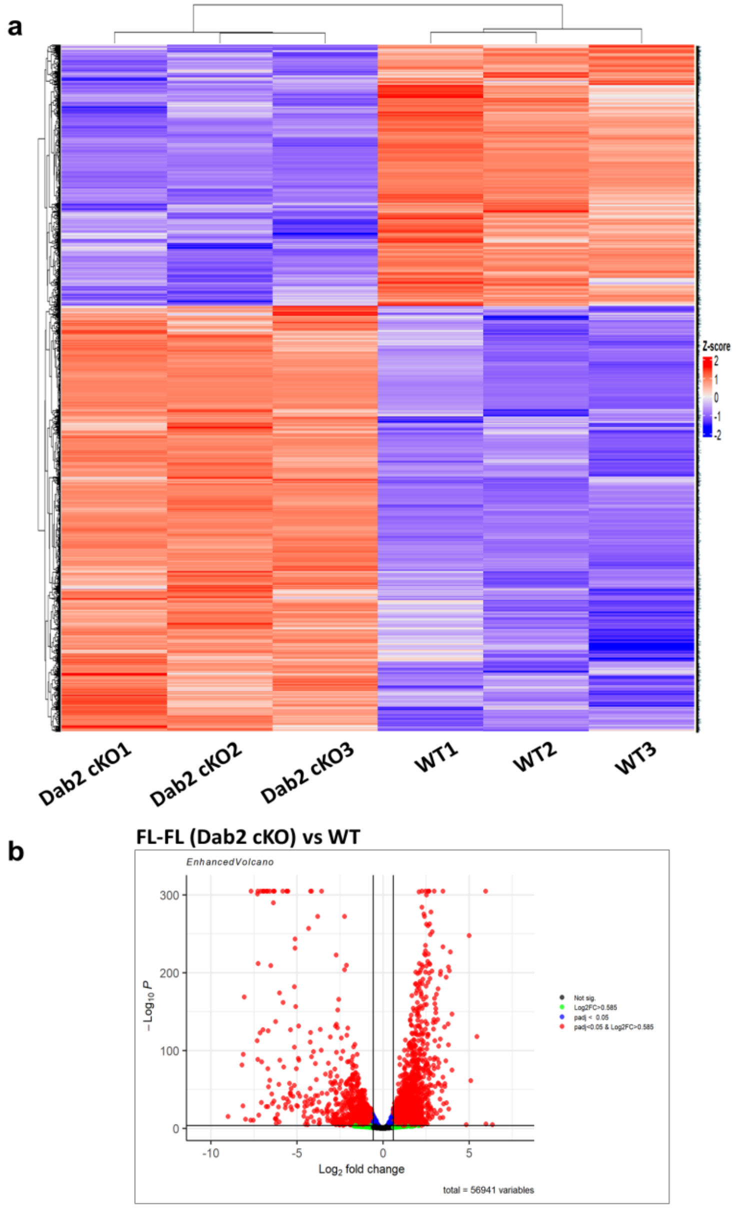

Supplementary Figure 8: Dab2 loss results in differential expression of genes at PD68 (2nd telogen)

a) Heatmap showing DEGs with p adj > 0.05 in FACS sorted WT and Dab2-cKO HFSCs at PD68, n=3 independent biological replicates. The analysis was performed using the Complex Heatmap R package version 2.18.0.

b) DEGs were identified from the count data using the DESeq2 package version1.42.0. The volcano plot of the DESeq2 output was plotted using the EnhancedVolcano R package version 1.20.0.

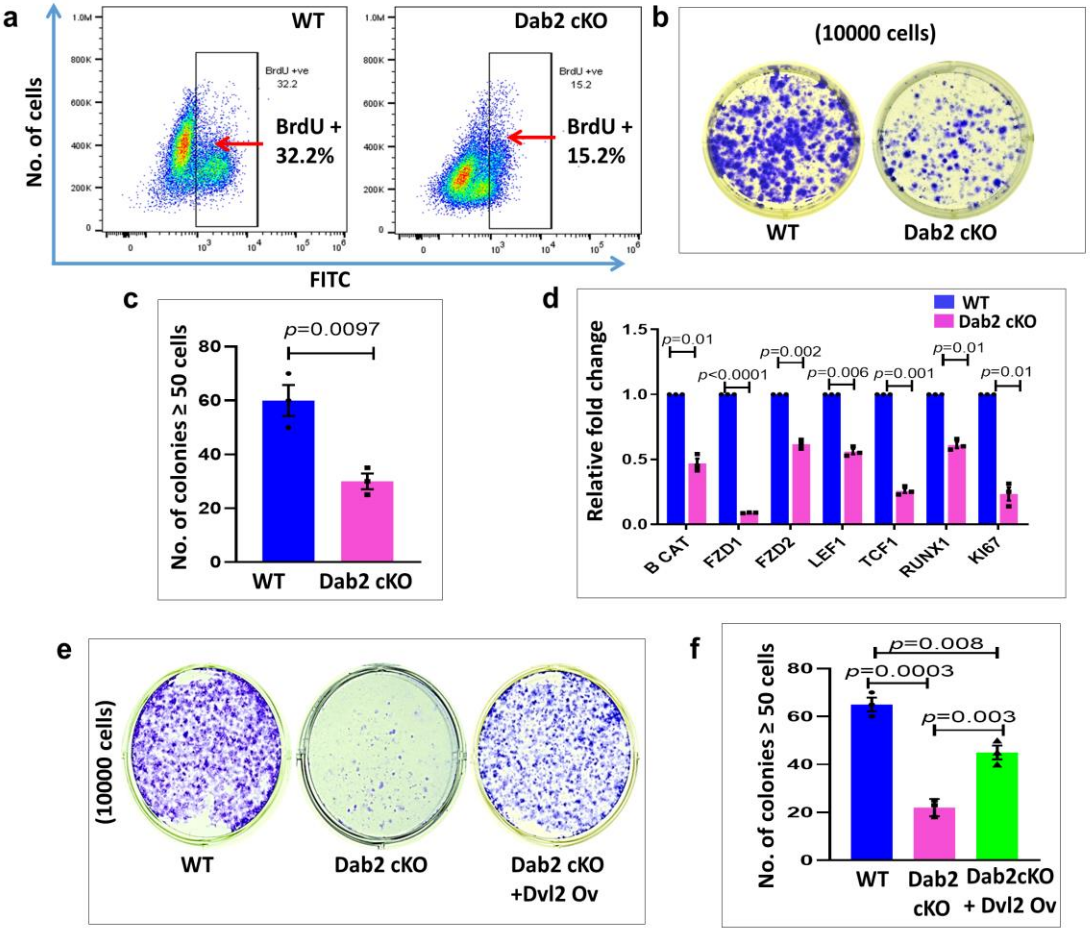

Supplementary Figure 9: Effect of Dab2 loss on murine primary keratinocytes

a) FACS analysis of BrdU+ cells in synchronized WT and Dab2-cKO primary keratinocytes, n=3 independent experimental replicates for each genotype.

b) Colonies formed by 10000 WT and Dab2-cKO primary keratinocytes after 2 weeks of culture, n=3 independent experimental replicates for each genotype.

c) Graph showing number of colonies formed by WT and Dab2-cKO primary keratinocytes after 2 weeks of culture, n=3 independent experimental replicates.

d) qPCR analysis of Wnt pathway genes expression in WT and Dab2-cKO primary keratinocytes, n=3 independent experimental replicates for each genotype.

e) Colonies formed by 10000 WT, Dab2-cKO and Dab2-cKO+Dvl2 overexpressed primary keratinocytes after 2 weeks of culture, n=3 independent experimental replicates.

f) Graph showing number of colonies formed by WT, Dab2-cKO & Dab2-cKO+Dvl2 overexpressed primary keratinocytes after 2 weeks of culture, n=3 independent experimental replicates.

(Data represent mean ± SEM, * = $p<0.05$, ** = $p<0.01$, *** = $p<0.001$, **** = $p<0.0001$ obtained by students t-test).

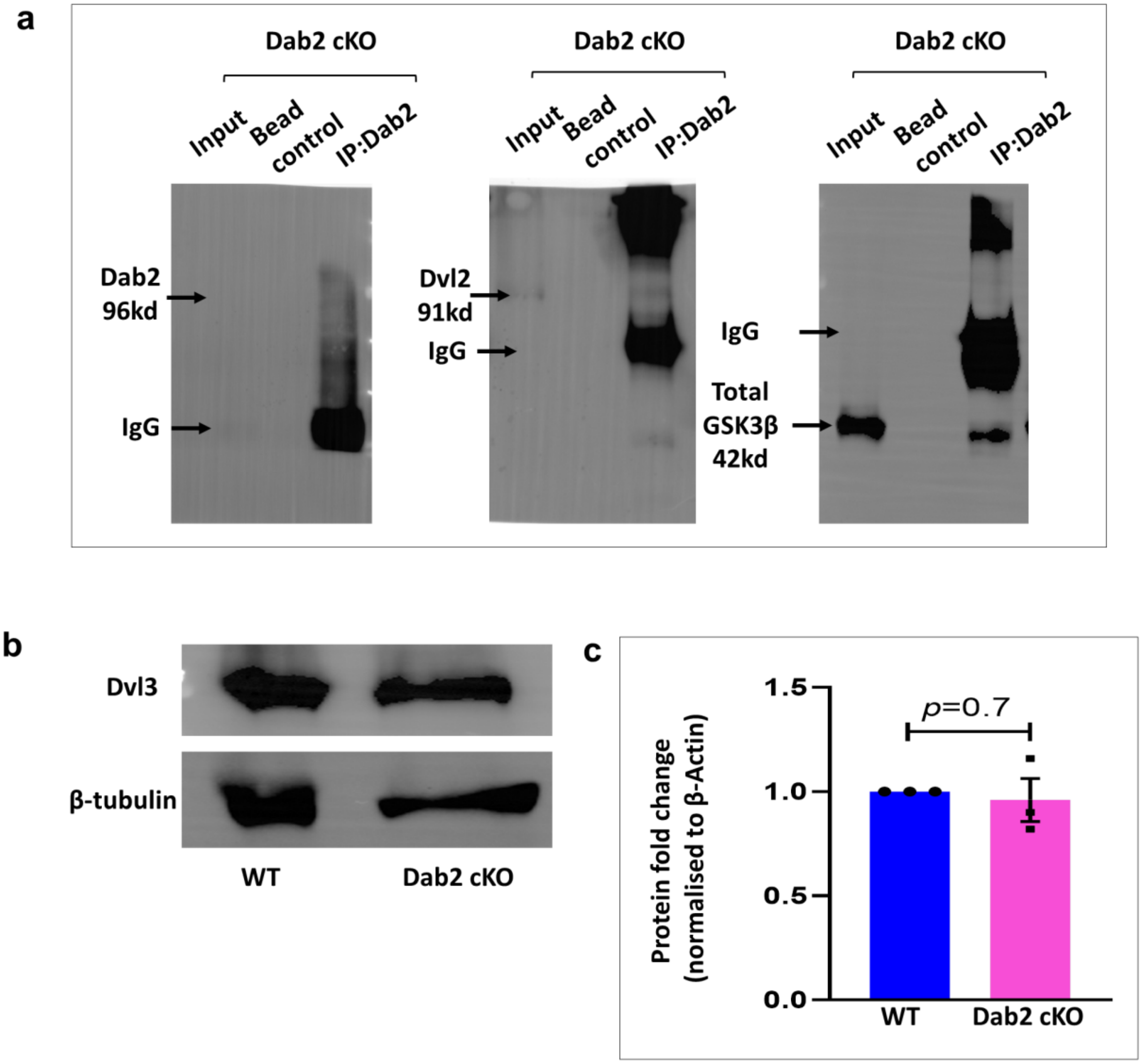

Supplementary Figure 10: Co-IP studies in Dab2-cKO primary keratinocytes

a) Co-IP of Dab2 with Dab2, Dvl2 and Total GSK3β immunoblotting in Dab2-cKO primary keratinocytes, n=3 independent experimental replicates.

b) Immunoblot analysis of Dvl3 in WT and Dab2-cKO primary keratinocytes, n=3 independent experimental replicates for each genotype.

c) Graph showing relative fold change of Dvl3 in WT and Dab2-cKO primary keratinocytes, n=3 independent experimental replicates for each genotype.

(Data represent mean ± SEM, * = p<0.05, ** = p<0.01, *** = p<0.001, **** = p<0.0001 obtained by students t-test).

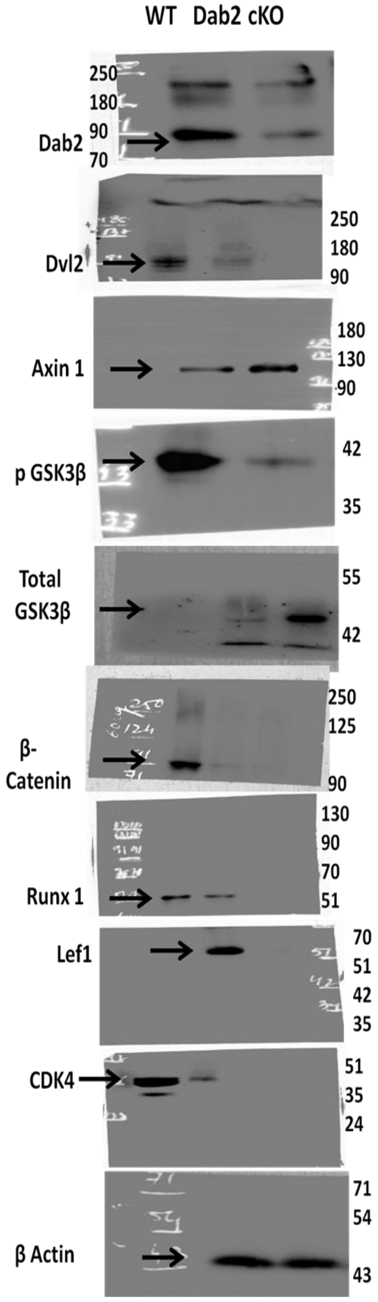

Supplementary Figure 11: Uncropped western blot images corresponding to Figure 7f

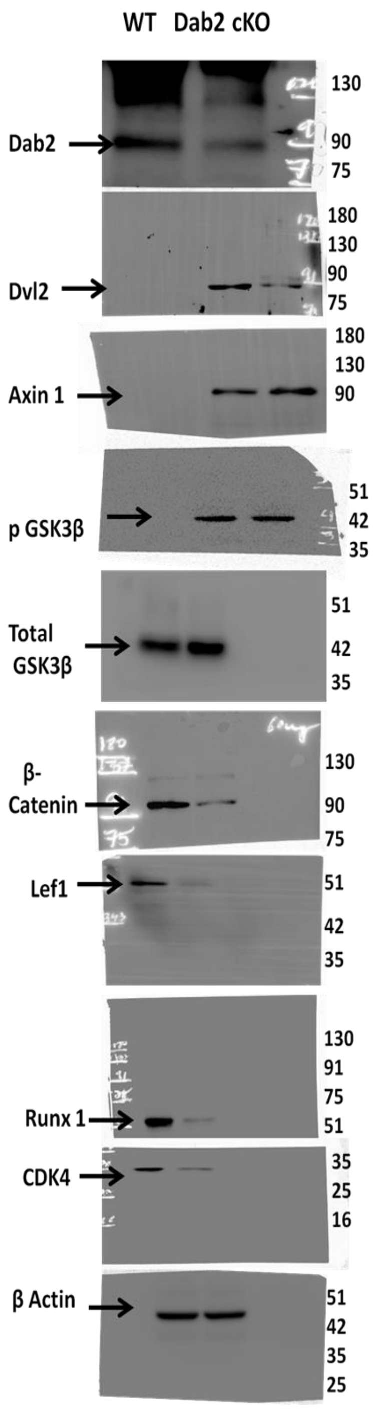

Supplementary Figure 12: Uncropped western blot images corresponding to Figure 8c

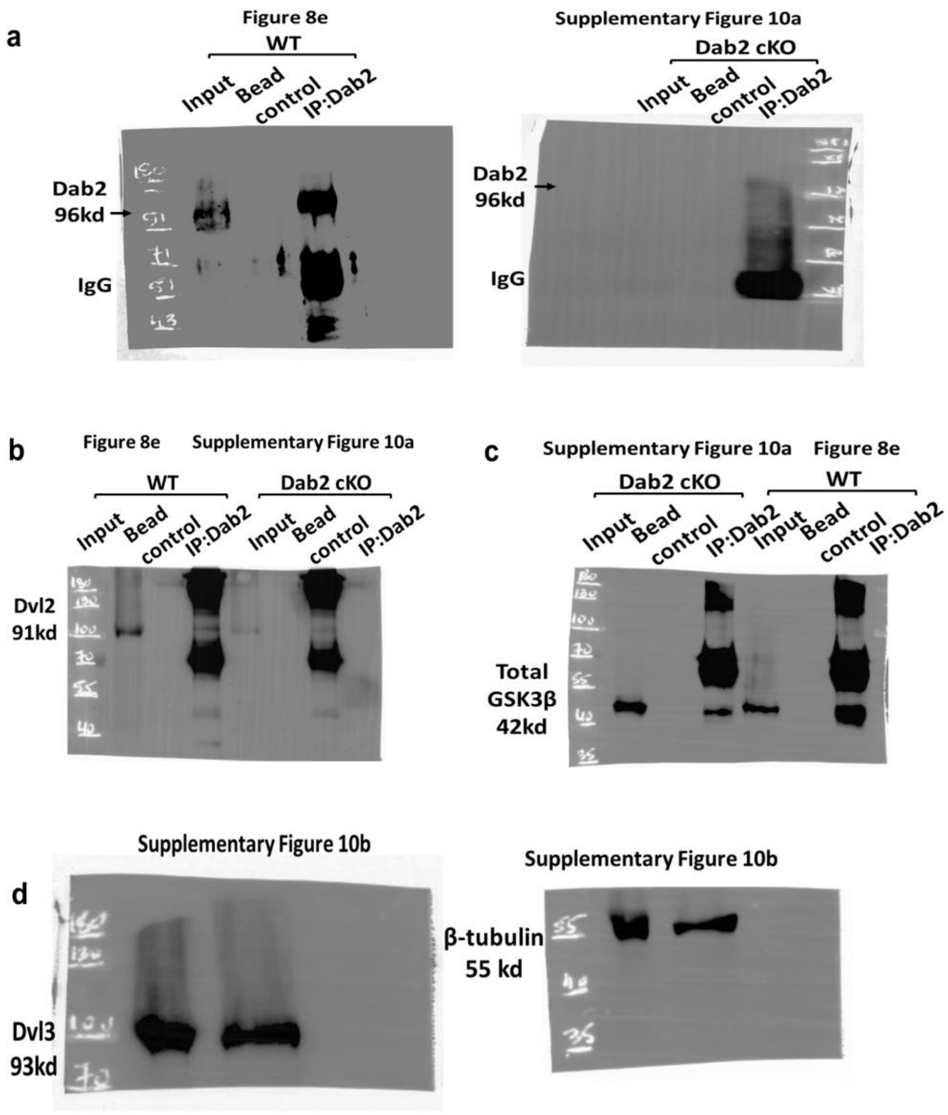

Supplementary Figure 13: Uncropped western blot images

a) Uncropped Co-IP blots of Dab2 with Dab2 immunoblotting in WT and Dab2-cKO primary keratinocytes corresponding to the figures as indicated.

b) Uncropped Co-IP blots of Dab2 with Dvl2 immunoblotting in WT and Dab2-cKO primary keratinocytes corresponding to the figures as indicated.

c) Uncropped Co-IP blots of Dab2 with Total GSK3β immunoblotting in WT and Dab2-cKO primary keratinocytes corresponding to the figures as indicated.

d) Uncropped western blot images of Dvl3 and β-tubulin in WT and Dab2-cKO primary keratinocytes corresponding to the figures as indicated.
