# Supplementary material for: Dab2 (Disabled-2), an adaptor protein, regulates self-renewal of hair follicle stem cells
Source: Commun Biol. 2024 May 3;7:525. doi: 10.1038/s42003-024-06047-2 (PMC11068889; doi:10.1038/s42003-024-06047-2)
Supplement: Supplementary file 2 — Description of Additional Supplementary Files [file 42003_2024_6047_MOESM2_ESM.pdf]

## Description of Additional Supplementary Files

**File name:** Supplementary Data 1

**Description:** The source data behind the graphs in the paper

**File name:** Supplementary Data 2

**Description:** FACS staining strategies

**File name**: Supplementary Data 3

**Description:** FACS gating strategy

**File name:** Supplementary Data 4

**Description:** List of primers and antibodies.
