# Supplementary material for: Dab2 (Disabled-2), an adaptor protein, regulates self-renewal of hair follicle stem cells
Source: Commun Biol. 2024 May 3;7:525. doi: 10.1038/s42003-024-06047-2 (PMC11068889; doi:10.1038/s42003-024-06047-2)
Supplement: Supplementary file 3 — Supplementary Data 1-4 [file 42003_2024_6047_MOESM3_ESM.zip › Supplementary Data 2.pdf]

Supplementary Data 2: FACS staining strategy

<table>
<tr><th>Tubes</th><th>Step 1</th><th>Step 2</th><th>Step 3</th><th>Step 4</th><th>Step 5</th></tr>
<tr><td></td><td>Resuspending the cells in each tube and addition of antibodies</td><td rowspan="9">a) Add the antibodies in the required tubes and mix well by gentle tapping. Incubate the tubes at 4ºC for 30 minutes. Do not allow cells to settle down and form clumps during the incubation period,

b) Add double volume of ice cold 1X PBS in the tube no 6 and 7.

c) Centrifuge the tubes no 6 and 7 at 1000 rpm for 5 minutes at 4ºC.

d) Discard the supernatant and add 100 µl FACS buffer in tube no. 6 and 750 µl in tube no 7.</td><td></td><td rowspan="9">a) Mix well and incubate the tubes at 40C for 30 min. keep tapping in between.

b) Add double the volume of ice cold 1X PBS in all the tubes after incubation.

c) Centrifuge the tubes at 1000 rpm for 5 min. at 40C.

d) Meanwhile, prepare PI in FACS buffer at a final concentration of 50µg/ml.

e) Discard the supernatant and resuspend the pellet as shown in the following steps</td><td>Resuspending the cells in each tube</td></tr>
<tr><td></td><td></td><td></td><td></td></tr>
<tr><td>Only cells</td><td>100 µl FACS buffer</td><td>-----</td><td>-----</td></tr>
<tr><td>PI control</td><td>100 µl FACS buffer</td><td>-----</td><td>Resuspend the cells in 100µl of PI solution</td></tr>
<tr><td>Iso PE</td><td>100 µl FACS buffer</td><td>1.3 µl Iso PE</td><td>100 µl FACS buffer</td></tr>
<tr><td>α6 PE</td><td>100 µl FACS buffer</td><td>2.6 µl α6 PE</td><td>100 µl FACS buffer</td></tr>
<tr><td>Iso APC</td><td>100 µl FACS buffer</td><td>1 µl Streptavidin-APC Antibody</td><td>100 µl FACS buffer</td></tr>
<tr><td>CD34 +APC</td><td>100 µl FACS buffer +
2 µl of CD34 Antibody</td><td>1 µl Streptavidin-APC Antibody</td><td>100 µl FACS buffer</td></tr>
<tr><td>Test</td><td>750ul FACS Buffer +
15 µl of CD34 Antibody</td><td>7.5 µl Streptavidin-APC Antibody</td><td>750µl PI solution</td></tr>
</table>
