# Supplementary material for: Dab2 (Disabled-2), an adaptor protein, regulates self-renewal of hair follicle stem cells
Source: Commun Biol. 2024 May 3;7:525. doi: 10.1038/s42003-024-06047-2 (PMC11068889; doi:10.1038/s42003-024-06047-2)

Supplementary Data 3:

a) Gating strategy for analysis of CD34+/α6+ HFSC population by FACS

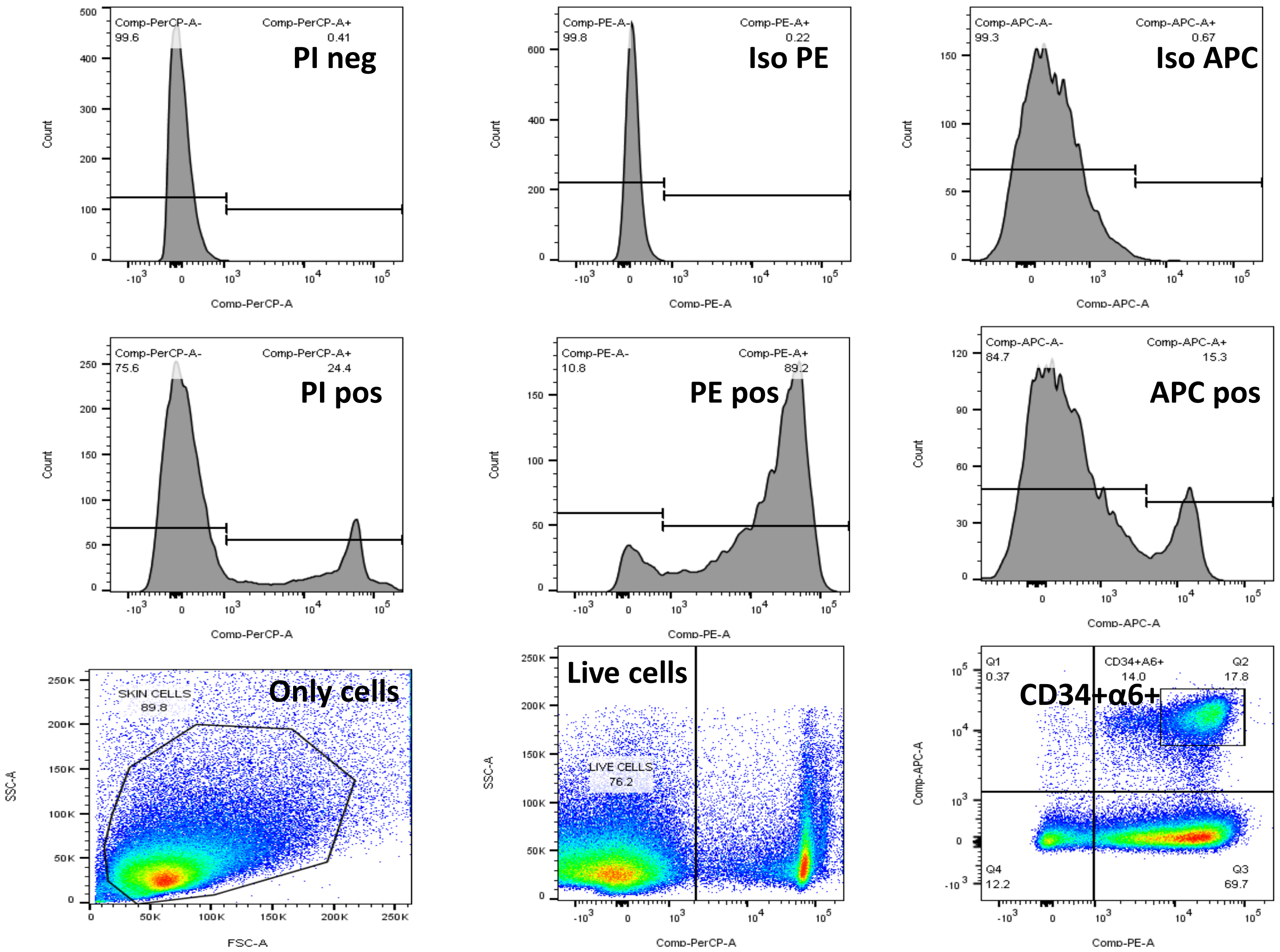

b) Proliferation dynamics study gating strategy

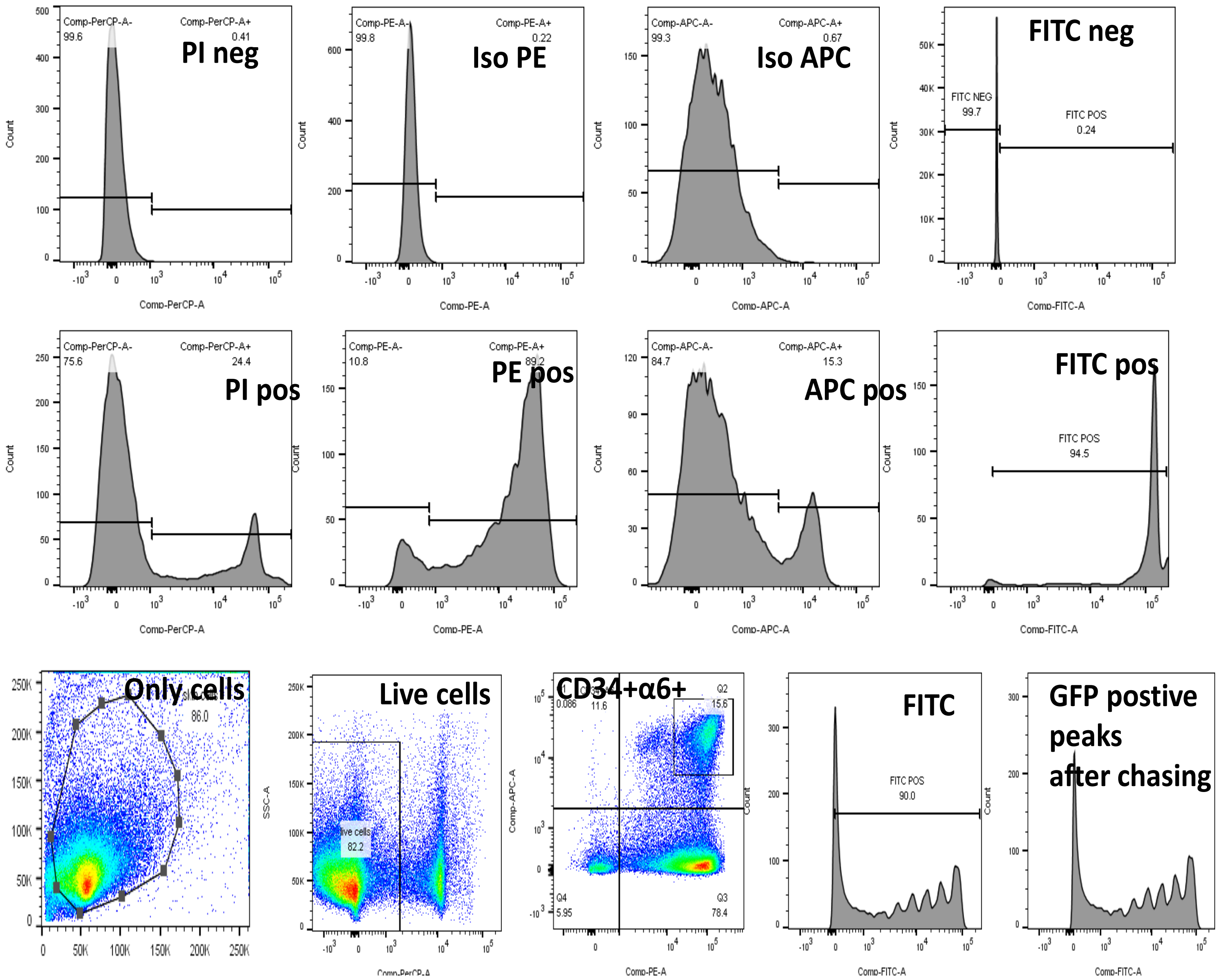

Supplement: Supplementary file 3 — Supplementary Data 1-4 [file 42003_2024_6047_MOESM3_ESM.zip › Supplementary Data 3.pdf]
