# Supplementary material for: Dab2 (Disabled-2), an adaptor protein, regulates self-renewal of hair follicle stem cells
Source: Commun Biol. 2024 May 3;7:525. doi: 10.1038/s42003-024-06047-2 (PMC11068889; doi:10.1038/s42003-024-06047-2)
Supplement: Supplementary file 3 — Supplementary Data 1-4 [file 42003_2024_6047_MOESM3_ESM.zip › Supplementary Data 4.pdf]

Supplementary Data 4:

a) List of antibodies

Antibody for IF	Company	Catalogue No.	Dilution for IF	Dilution for WB
Dab2	BD transduction Laboratories	610465	1:75	1:1000
CD34	BD Pharmingen	553731	1:100	-
K15	Abcam	ab52816	1:200	-
Sox9	CST	#82630	1:100	-
NFATc1	Santa cruz	sc-7294	1:75	-
BrdU	Abcam	ab6326	1:100	-
Ki67	Abcam	ab15580	1:100	1:75
Lef1	CST	#2230	1:200	1:1000
β-Cat	CST	#8480	1:200	1:1000
Axin1	CST	#2087	1:100	1:1000
GSK3β	CST	#12456	1:100	1:1000
Dvl2	CST	#3224	1:100	1:1000
Dab2 (Co-IP)	CST	#12906	-	1:1000
CDK4	Santa Cruz	sc-23896	-	1:1000
Runx1	Santa Cruz	sc365644	-	1:1000
pGSK3β	CST	#9323	-	1:1000
β- Actin	CST	#8457	-	1:2000
β- Tubulin	CST	#12146		1:2000
Anti-GFP	Abcam	ab13970	1:200	-
Anti-Rabbit 488	Abcam	ab150077	1:200	-
Anti- Rabbit Alexa Fluor 568	Abcam	ab175471	1:400	-
Anti mouse Alexa Fluor 488	Jackson Immuno-research	115-095-003	1:200	-
Anti-mouse Cy3	Jackson Immuno-research	115-165-003	1:400	-
Anti Rat FITC	Jackson Immuno-research	112-095-003	1:400	-
Anti Rat Alexa Fluor 568	Abcam	ab175476	1:400	
Anti-Rabbit IgG HRP linked	CST	#7074	-	1:2000- 1:4000
Anti-Mouse IgG HRP linked	CST	#7076	-	1:2000- 1:4000

Antibody for FACS	Company	Catalogue No.	Dilution for FACS
Anti mouse CD34 Biotin	eBiosciences	13-0341	15µl/ reaction for test, 2 µl for control
APC streptavidin	BD pharmingen	554067	7.5µl/reaction for test, 1µl for controls
PE Rat α-6 integrin (CD49f) PE	BD biosciences	555734	20µl/ reaction for test, 2.6µl for positive control
PE Rat isotype control	BD pharmingen	555844	1.3ul/ reaction for isotype control
Anti BrdU FITC	BD Biosciences	347583	20µl/ reaction for test

b) List of primer sequences

Primer name	Sequence
K14 Cre Forward	GCGGTCTGGCAGTAAAAACTATC
K14 Cre Reverse	GTGAAACAGCATTGCTGTCACTT
K14 IC Forward	CTAGGCCACAGAATTGAAAGATCT
K14 IC Reverse	GTAGGTGGAAATTCTAGCATCATCC
DAB2 FLOX Forward	TTGATGATGTGCCTGATGCT
DAB2 FLOX Reverse	AAGAGAACACTGGAGGCTCA
K5TTA Forward	CTCGCCCAGAAGCTAGGTGT
K5TTA R	CCATCGCGATGACTTAGTAA
GFP Forward	AAGTTCATCTGCACCACCG
GFP R	TCCTTGAAGAAGATGGTGCG
ROSA LacZ mutantR	GCGAAGAGTTTGTCCTCAACC
ROSA LacZ common	AAAGTCGCTCTGAGTTGTTAT
ROSA LacZ wildtypeR	GGAGCGGGAGAAATGGATATG
ROSA YFP mutantR	AAGACCGCGAAGAGTTTGTC
ROSA YFP common	AAAGTCGCTCTGAGTTGTAT
ROSA YFP wildtypeR	GGAGCGGGAGAAATGGATATG
DAB2 MOUSE Forward	TGTGGGTTCTGTCCTTTTGAG
DAB2 MOUSE Reverse	CTTCTTCTCCTTCTTTGATGGC
B ACTIN Forward	CTAAGGCCAACCGTGAAAAG
B ACTIN Reverse	ACCAGAGGCATACAGGGACA
NFATC1 Forward	GGTCCCTATCAAGTCTCG
NFATC1 Reverse	AGGTGCTGGAAGGTGTA
Ki67 Forward	AGGAGGAACCAACCAAGGACAGTT
Ki67 Reverse	TTTCTCTGTGCTGTGGGCTCTTCT
COL17A Forward	AAGTCACCGAGAGAATTGTCAC
COL17A Reverse	AGAGAGCCTGTCTTAGCATATCC
DECORIN Forward	TCTTGGGCTGGACCATTTGAA
DECORIN R c-myc	CATCGGTAGGGGCACATAGA
SIRT2 Forward	GAGCCGGACCGATTCAGAC
SIRT2 Reverse	AGACGCTCCTTTTGGGAACC

SIRT7 Forward	CTAAGCGAAGCGGAGCCTAC
SIRT7 Reverse	GTGGAGCCCATCACAGTTCT
S100A4 Forward	TCCACAAATACTCAGGCAAAGAG
S100a4 Reverse	GCAGCTCCCTGGTCAGTAG
Runx1 Forward	GAGATTCAACGACCTCAGGTTT
Runx1 Reverse	TGTAAAGACGGTGATGGTCAGA
Cyclind1 Forward	TGACTGCCGAGAAGTTGTGC
Cyclind1 Reverse	CTCATCCGCCTCTGGCATT
B CAT Forward	CCCAGTCCTTCACGCAAGAG
B CAT Reverse	CATCTAGCGTCTCAGGGAACA
LEF1 Forward	GCCACCGATGAGATGATCCC
LEF1 Reverse	TTGATGTCGGCTAAGTCGCC
TCF4 Forward	CAAGCACTGCCGACTACAATA
TCF4 Reverse	CCAGGCTGATTCATCCCACTG
TCF1 Forward	CCAGTGTGCACCCTTCCTAT
TCF1 Reverse	AGCCCCACAGAGAAACTGAA
FZD1 Forward	AACTTTGTGCCGAAGCACTC
FZD1 Reverse	GGTCTGGTTGTACGCGATGT
GSK3B Forward	AAGCGATTTAAGAACCGAGAGC
GSK3B Reverse	AGAAATACCGCAGTCGGACTAT
SFRP4 Forward	AGAAGGTCCATACAGTGGGAAG
SFRP4 Reverse	GTTACTGCGACTGGTGCGA
DKK1 Forward	ATATCACACCAAAGGACAAGAAGG
DKK1 Reverse	AGGTTTACAGATCTTGGACCAGAA
DKK2 Forward	CTGATGCGGGTCAAGGATTCA
DKK2 Reverse	CTCCCCTCCTAGAGAGGACTT
DKK3 Forward	AGCTGCTAAAACGTCCTCTGA
DKK3 Reverse	CTGGTCTCATTGTGATAGTTGGG
Wnt3a Forward	AATTTGGAGGAATGGTCTCTCGG
Wnt3a Reverse	CAGCAGGTCTTCACTTCACAG
Wnt5b Forward	CCAGTGCAGAGACCGGAGATG
Wnt5b Reverse	GTTGTCCACGGTGCTGCAGTTC
Wnt7B Forward	ATCGCATTTTCTCGTCGCTTT
WNT7B Reverse	ATCGACTTTTCTCGTCGCTTT
WNT 5A Forward	AGCCTGTAAGTGTCATGGAGT
WNT 5A Reverse	CGCGGCGCTATCATACTTCT
P21 Forward	CGCTGTCTTGCACTCTGGT
P21 Reverse	CGTTTTCGGCCCTGAGATGTT
c-myc Forward	CAGCGACTCTGAAGAAGAGC
c-myc Reverse	GTTGTGCTGGTGAGTGGAGA
