# Supplementary material for: Dab2 (Disabled-2), an adaptor protein, regulates self-renewal of hair follicle stem cells
Source: Commun Biol. 2024 May 3;7:525. doi: 10.1038/s42003-024-06047-2 (PMC11068889; doi:10.1038/s42003-024-06047-2)
Supplement: Supplementary file 4 — Reporting Summary [file 42003_2024_6047_MOESM4_ESM.pdf]

nature portfolio

Corresponding author(s): Dr. Sanjeev K Waghmare

Last updated by author(s): Mar 1, 2024

Reporting Summary

Nature Portfolio wishes to improve the reproducibility of the work that we publish. This form provides structure for consistency and transparency in reporting. For further information on Nature Portfolio policies, see our Editorial Policies and the Editorial Policy Checklist.

Statistics

For all statistical analyses, confirm that the following items are present in the figure legend, table legend, main text, or Methods section.

n/a	Confirmed	
☐	☒	The exact sample size (*n*) for each experimental group/condition, given as a discrete number and unit of measurement
☐	☒	A statement on whether measurements were taken from distinct samples or whether the same sample was measured repeatedly
☐	☒	The statistical test(s) used AND whether they are one- or two-sided<br>*Only common tests should be described solely by name; describe more complex techniques in the Methods section.*
☒	☐	A description of all covariates tested
☒	☐	A description of any assumptions or corrections, such as tests of normality and adjustment for multiple comparisons
☐	☒	A full description of the statistical parameters including central tendency (e.g. means) or other basic estimates (e.g. regression coefficient) AND variation (e.g. standard deviation) or associated estimates of uncertainty (e.g. confidence intervals)
☒	☐	For null hypothesis testing, the test statistic (e.g. *F*, *t*, *r*) with confidence intervals, effect sizes, degrees of freedom and *P* value noted<br>*Give P values as exact values whenever suitable.*
☒	☐	For Bayesian analysis, information on the choice of priors and Markov chain Monte Carlo settings
☒	☐	For hierarchical and complex designs, identification of the appropriate level for tests and full reporting of outcomes
☒	☐	Estimates of effect sizes (e.g. Cohen's *d*, Pearson's *r*), indicating how they were calculated

Our web collection on statistics for biologists contains articles on many of the points above.

Software and code

Policy information about availability of computer code

Data collection	For IFA, Imaging was done using Zeiss LSM 780 Confocal microscope<br>For Flow Cytometry, samples were sorted using BD FACS Aria (BD Biosciences)<br>For qPCR, Quantstudio 12k flex real-time PCR (Applied Biosystems, USA) was used<br>For immunoblotting, Biorad chemidoc imaging system was used<br>Expression analysis was performed using Clariom D Assay, Mouse previously known as GeneChip MTA 1.0 array, Affymetrix, USA
Data analysis	For IFA, image analysis were done using Zen 2.3<br>For Flow Cytometry, samples were analysed using FlowJo software<br>For qPCR , statistical analysis was performed using Graph Pad Prism 8<br>For Immunoblotting, densitometric analysis were performed using Quantity One 4.6.1<br>Expression analysis was performed using Affymetrix Trancriptome Analysis Console (TAC) software<br>RNA sequencing analysis was performed using the ComplexHeatmap R package version 2.18.0. The DEGs were identified from the count data using the DESeq2 package version1.42.0. The volcano plot of the DESeq2 output was plotted using the Enhanced Volcano R package version 1.20.0.

For manuscripts utilizing custom algorithms or software that are central to the research but not yet described in published literature, software must be made available to editors and reviewers. We strongly encourage code deposition in a community repository (e.g. GitHub). See the Nature Portfolio guidelines for submitting code & software for further information.

Data

Policy information about availability of data

All manuscripts must include a data availability statement. This statement should provide the following information, where applicable:

- Accession codes, unique identifiers, or web links for publicly available datasets
- A description of any restrictions on data availability
- For clinical datasets or third party data, please ensure that the statement adheres to our policy

A data availability statement has been provided in the manuscript.
The expression profile data of HFSCs of WT and Dab2 cKO at PD35 have been submitted to the GEO database bearing the accession number GSE250300.
The expression profile (RNA seq.) of HFSCs of WT and Dab2 cKO at PD68 have been submitted to the GEO database bearing the accession number GSE254311.
The source data for all the graphs has been provided in the Supplementary information

Research involving human participants, their data, or biological material

Policy information about studies with human participants or human data. See also policy information about sex, gender (identity/presentation), and sexual orientation and race, ethnicity and racism.

Reporting on sex and gender	Our study does not involve the use of human participants
Reporting on race, ethnicity, or other socially relevant groupings	Our study does not involve this issue
Population characteristics	Our study does not involve this issue
Recruitment	Our study does not involve this issue
Ethics oversight	Our study does not involve this issue

Note that full information on the approval of the study protocol must also be provided in the manuscript.

Field-specific reporting

Please select the one below that is the best fit for your research. If you are not sure, read the appropriate sections before making your selection.

☒ Life sciences ☐ Behavioural & social sciences ☐ Ecological, evolutionary & environmental sciences

For a reference copy of the document with all sections, see nature.com/documents/nr-reporting-summary-flat.pdf

Life sciences study design

All studies must disclose on these points even when the disclosure is negative.

Sample size	Samples size for each experiment is indicated in the corresponding figure legends. No statistical methods were used to predetermine sample size
Data exclusions	No data were excluded for analysis
Replication	All the experiments were performed in at least 3 independent biological replicates. Statistics were performed on biological replicates and not on technical replicates
Randomization	For all in vivo studies, mice were grouped based on genotypes: K14 CreER-/- Dab2 fl/fl (WT), K14 CreER+/- Dab2 fl/fl (Dab2 cKO). Only female mice were used for experimental purposes. Tamoxifen (75mg/kg/day) was injected intraperitoneally in both the groups. Similarly, for in vitro studies keratinocytes obtained from female neonatal mice at PD2 were grouped into 2 genotypes: K14 CreER-/- Dab2 fl/fl (WT), K14 CreER+/- Dab2 fl/fl (Dab2 cKO). After successful generation of primary keratinocytes, both the WT and Dab2 cKO were treated with 4-Hydroxytamoxifen (4-OHT, 1um) for 5 consecutive days. E-Media containing 4-OHT was replaced after every 48 hrs till 5 days . After 5 days, the cells were maintained in normal E-media
Blinding	Investigators were not blinded to mouse genotypes or cell lines genotypes during data collection and analysis

Reporting for specific materials, systems and methods

We require information from authors about some types of materials, experimental systems and methods used in many studies. Here, indicate whether each material, system or method listed is relevant to your study. If you are not sure if a list item applies to your research, read the appropriate section before selecting a response.

Materials & experimental systems

n/a	Involved in the study
☐	☒ Antibodies
☐	☒ Eukaryotic cell lines
☒	☐ Palaeontology and archaeology
☐	☒ Animals and other organisms
☒	☐ Clinical data
☒	☐ Dual use research of concern
☒	☐ Plants

Methods

n/a	Involved in the study
☒	☐ ChIP-seq
☐	☒ Flow cytometry
☒	☐ MRI-based neuroimaging

Antibodies

Antibodies used

For Western and IF, the following antibodies have been used:

Dab2 BD transduction Laboratories 610465
CD34 BD Pharmingen 553731
K15 Abcam ab52816
Sox9 CST #82630
NFATc1 Santa cruz sc-7294
BrdU Abcam ab6326
Ki67 Abcam ab15580
Lef1 CST #2230
β-Cat CST #8480
Axin1 CST #2087
GSK3β CST #12456
Dvl2 CST #3224
Dab2 (Co-IP) CST #12906
CDK4 Santa Cruz sc-23896
Runx1 Santa Cruz sc365644
pGSK3β CST #9323
β- Actin CST #8457
β- Tubulin CST #12146
Anti-GFP Abcam ab13970
Anti-Rabbit 488 Abcam ab150077
Anti- Rabbit Alexa Fluor 568 Abcam ab175471
Anti mouse Alexa Fluor 488 Jackson Immuno-research 115-095-003
Anti-mouse Cy3 Jackson Immuno-research 115-165-003
Anti Rat FITC Jackson Immuno-research 112-095-003
Anti Rat Alexa Fluor 568 Abcam ab175476
Anti-Rabbit IgG HRP linked CST #7074
Anti-Mouse IgG HRP linked CST #7076

For Flow Cytometry, following antibodies were used:

Anti mouse CD34 Biotin eBiosciences 13-0341
APC streptavidin BD pharmingen 554067
PE Rat α-6 integrin (CD49f) PE BD biosciences 555734
PE Rat isotype control BD pharmingen 555844
Anti BrdU FITC BD Biosciences 347583

A list containing the dilutions used for all the anibodies has been provided in the Supplementary information.

Validation

Most of the antibodies were used and validated in the previous manuscripts from our lab (Raghava S. et al, Stem cells, 2022), (Gopal C.et al, Ebiomedicine, 2019), (Rahul S et al, Stem Cells, 2016). The Dab2 antibodies were validated according to the manufacturer's official website information.

Eukaryotic cell lines

Policy information about cell lines and Sex and Gender in Research

Cell line source(s)	Primary keratinocytes were obtained from female neonatal mouse pups at PD2 in our lab
Authentication	None of the cell lines have been authenticated
Mycoplasma contamination	Cell lines were not checked for mycoplasma contamination
Commonly misidentified lines (See ICLAC register)	None of the cell lines used are listed in the database of commonly misidentified cell lines maintained by ICLAC

Animals and other research organisms

Policy information about studies involving animals; ARRIVE guidelines recommended for reporting animal research, and Sex and Gender in Research

Laboratory animals	K14 CreER+/- and Dab2 fl fl were imported from Jackson laboratories. PreH2BGFP and K5tta were procured from Jackson, USA and Prof Rune Toftgard, Sweden respectively. Mice were bred, crossed and maintained at ACTREC Animal House facility under pathogen free conditions
Wild animals	No wild animals were used for our study
Reporting on sex	Only females were used for our experiments
Field-collected samples	All mice were maintained under dark & light cycles and controlled conditions for temperature, humidity, air pressure as per the regulatory requirements with the help of HVAC system.
Ethics oversight	All animal experiments were approved by ACTREC Institutional Animal Ethics Committee. Our institutes follow CPSEA guidelines provided by India's Ministry of Environment and Forests government for all animal experiments. We have complied with all relevant ethical regulations for animal use. IAEC project number 04/2022 were approved in the April 2022.

Note that full information on the approval of the study protocol must also be provided in the manuscript.

Flow Cytometry

Plots

Confirm that:

☒ The axis labels state the marker and fluorochrome used (e.g. CD4-FITC).

☒ The axis scales are clearly visible. Include numbers along axes only for bottom left plot of group (a 'group' is an analysis of identical markers).

☒ All plots are contour plots with outliers or pseudocolor plots.

☒ A numerical value for number of cells or percentage (with statistics) is provided.

Methodology

Sample preparation	Mice were sacrificed and skin was collected at the designated days. Excess fat was removed from the dermis side by scraping. Skin was washed with 1X PBS and placed in the 90 mm plate containing 10 ml of the ice-cold 0.25% Trypsin-EDTA solution. Plates were incubated in 4°C refrigerator O/N. Next day, fresh 10 ml of the 0.25% ice-cold Trypsin-EDTA solution was added in all the plates containing skin and incubated at 37°C for 30 minutes. Trypsin was neutralized by adding 30 ml cold E-Media containing 15% chelated FBS. Epidermis was scraped off from the dermis and minced with the help of a blade.<br>The solution was passed through glass pipette multiple times to make single cell suspension that was further strained using 70 µm and 40 µm strainers. The filtered solution was centrifuged at 2000 rpm for 5 minutes at 4°C. The cell pellet was washed by using ice-cold 1X PBS and centrifuged again. The cell pellet was then dissolved in 750 µl of 5% chelated FBS in 1X PBS (FACS buffer) and stained with the required antibodies.
Instrument	Facs Aria machine (BD Biosciences)
Software	FlowJo software
Cell population abundance	The sorting efficiency was checked by post sort efficiency and it was found to be 90%
Gating strategy	Forward scatter and side scatter were used for selecting entire cell population. Live cells were selected with the help of PI staining. CD34 APC and alpha 6-integrin PE were used for sorting hair follicle stem cells. For GFP chasing experiments, FITC positive CD34+/alpha 6-integrin+ cells were analysed.

☒ Tick this box to confirm that a figure exemplifying the gating strategy is provided in the Supplementary Information.
